# Midbrain Gene Screening Identifies a New Mesoaccumbal Glutamatergic Pathway and a Marker for Dopamine Cells Neuroprotected in Parkinson’s Disease

**DOI:** 10.1038/srep35203

**Published:** 2016-10-20

**Authors:** Thomas Viereckel, Sylvie Dumas, Casey J. A. Smith-Anttila, Bianca Vlcek, Zisis Bimpisidis, Malin C. Lagerström, Åsa Konradsson-Geuken, Åsa Wallén-Mackenzie

**Affiliations:** 1 Department of Organismal Biology/Comparative Physiology, Uppsala University, S-752 36 Uppsala, Sweden; 2 Department of Neuroscience, Uppsala University, S-751 24 Uppsala, Sweden; 3 Oramacell, 75006 Paris, France; 4 Department of Anatomy and Neuroscience, University of Melbourne, Victoria 3010, Australia

## Abstract

The ventral tegmental area (VTA) and substantia nigra pars compacta (SNc) of the midbrain are associated with Parkinson’s disease (PD), schizophrenia, mood disorders and addiction. Based on the recently unraveled heterogeneity within the VTA and SNc, where glutamate, GABA and co-releasing neurons have been found to co-exist with the classical dopamine neurons, there is a compelling need for identification of gene expression patterns that represent this heterogeneity and that are of value for development of human therapies. Here, several unique gene expression patterns were identified in the mouse midbrain of which *NeuroD6* and *Grp* were expressed within different dopaminergic subpopulations of the VTA, and *TrpV1* within a small heterogeneous population. Optogenetics-coupled *in vivo* amperometry revealed a previously unknown glutamatergic mesoaccumbal pathway characterized by *TrpV1-Cre*-expression. Human GRP was strongly detected in non-melanized dopaminergic neurons within the SNc of both control and PD brains, suggesting GRP as a marker for neuroprotected neurons in PD. This study thus unravels markers for distinct subpopulations of neurons within the mouse and human midbrain, defines unique anatomical subregions within the VTA and exposes an entirely new glutamatergic pathway. Finally, both TRPV1 and GRP are implied in midbrain physiology of importance to neurological and neuropsychiatric disorders.

Dopamine (DA) neurons of the ventral tegmental area (VTA) and the substantia nigra (SN) pars compacta (SNc) in the ventral midbrain are essential for regulation of cognitive, affective and locomotor-related activities^1^,^2^. Dysfunctional neurotransmission of the DA neurons in the VTA is correlated with neuropsychiatric disorders such as drug addiction, schizophrenia and mood disorders, while degeneration of SNc DA neurons is the primary cause of Parkinson’s disease (PD)^3^,^4^,^5^. None of these incapacitating disorders can be prevented or cured wherefore major efforts are aimed at deciphering the complexity of the VTA and SNc.

Gene expression analysis have identified a range of developmental and adult genes that are expressed at different levels in DA neurons of the VTA and SNc, respectively^6^,^7^,^8^,^9^,^10^,^11^,^12^,^13^,^14^,^15^. In addition, it was recently shown that various subtypes of DA neurons exist within the VTA and SNc that are joined by similar combinatorial expression patterns^16^. DA neurons are thus more heterogeneous than previously believed. An additional level of heterogeneity is provided by the findings that DA neurons are intermingled with excitatory neurons, characterized by expression of the *Vesicular glutamate transporter* 2 (*Vglut2*) gene, as well as inhibitory neurons and various co-releasing neurons^17^,^18^,^19^,^20^,^21^,^22^. Transgenics-based studies, including knock-out^23^,^24^,^25^,^26^ and optogenetics^27^,^28^,^29^,^30^,^31^,^32^,^33^,^34^,^35^ approaches, have demonstrated that VTA/SNc-derived glutamate and GABA interact with various aspects of DA neurotransmission, and hence that these newly discovered VTA/SNc populations are involved in similar brain processes as the DA neurons. However, it is far from resolved how this occurs and what impact it may have on cognitive, affective and motoric function. Adding another level of complexity, the different neuronal populations are not evenly distributed throughout the VTA and SNc. Subtypes of DA neurons, for example, are differentially spread^16^ and the same is true for glutamatergic neurons; while present in both VTA and SNc, *Vglut2*-expressing neurons are most prominent within the medial aspect of the VTA, and here, a minority of them co-express the *Tyrosine hydroxylase* (*Th*) gene and thereby have the ability to co-release DA^18^,^21^,^22^,^36^,^37^,^38^,^39^,^40^,^41^.

The anatomical heterogeneity is likely reflected in the complex functionality of the VTA and SNc. Yet, all studies targeting DAergic, glutamatergic, GABAergic or co-releasing neurons within the VTA or SNc have relied entirely on the use neurotransmitter-selective promoters, such as the *DA transporter* (*Dat*), *Th*, *Vglut2* and *Glutamic acid decarboxylase* (*Gad*) promoters, for driving transgenic expressions, none of which fully represent the spatial selectivity required or the recently disclosed heterogeneity^17^,^42^,^43^.

In light of the recent realization of the multitude of different neuronal cell types that co-exist in the ventral midbrain, there is a compelling need for identification of gene expression patterns that represent this anatomical and functional heterogeneity. Such patterns should prove useful both for improved resolution in animal models and for clinical purposes. To this end, an unbiased microarray approach followed through by quantitative histological validation was used to analyze gene expression patterns within the VTA and SNc. Several genes that exhibited unique expression patterns within subareas of the VTA and SN were identified, including genes restricted within subpopulations of the VTA, e.g. *NeuroD6*, *Grp* and *TrpV1. TrpV1-Cre* transgenic mice were selected for functional optogenetics within the midbrain, while the human counterparts of the genes identified in the mouse were assessed in human brain material, including post-mortem tissue derived from PD patients.

## Results

### Unbiased gene-screening identifies 8 genes of interest as putative VTA or SNc markers

**Figure 1:**
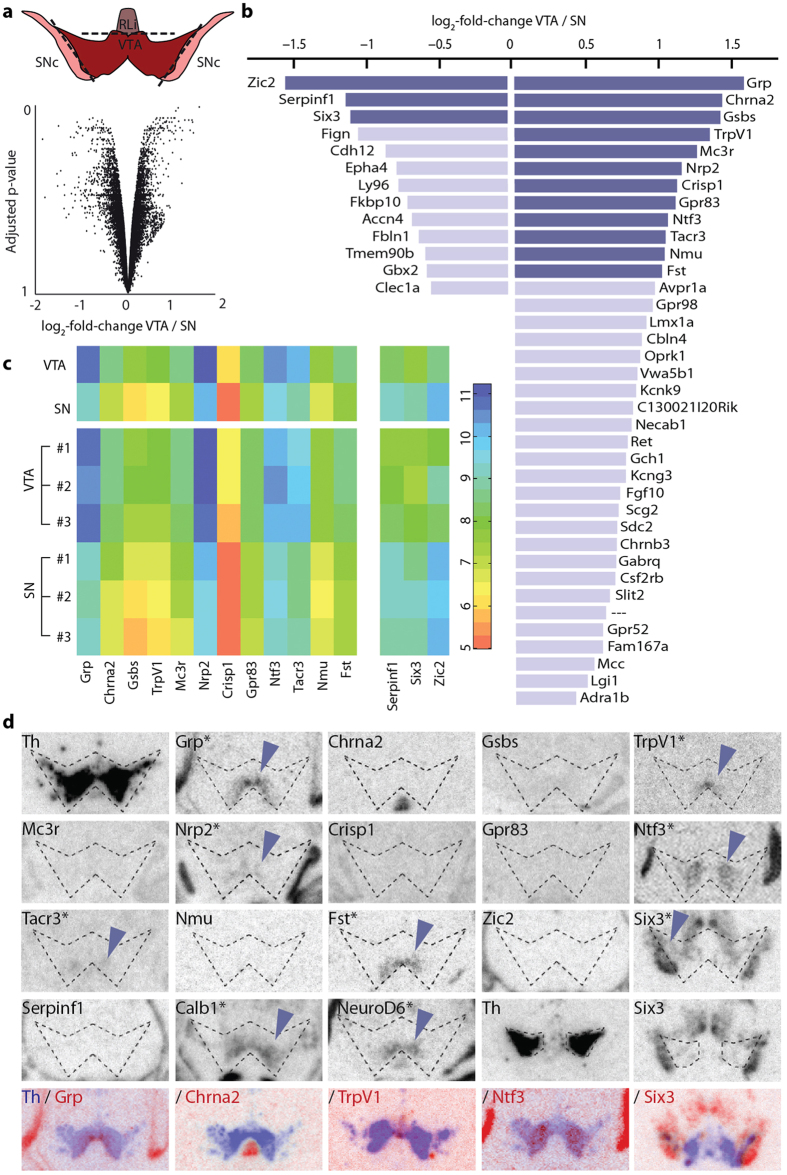
Identification of candidate genes with expression patterns restricted to either VTA or SNc. **(a**) Tissue obtained from P3 Dat-Cre^tdTom^ mice (n = 3) was separated into VTA, SNc and RLi (top) and VTA and SNc subjected to microarray analysis. Scatterplot of genes analyzed in the microarray, plotted by expression differences and adjusted p-value (bottom). Positive values represent genes upregulated in the VTA, negative values upregulation in the SNc. (**b)** Overview of the 50 genes with highest adjusted p-value, sorted according to difference in expression in VTA and SNc. Genes in dark blue were used for further analysis. (**c)** Heatmap of genes with at least log2-fold difference between VTA and SNc (n = 3) (top) and within the individual samples (bottom). (**d)** Representative images of oligo *in situ* hybridization for different genes in the ventral midbrain of P3 C57BL/6 mice with putative borders of the Th-positive cell population (dotted lines) as guidance. Rows one to three and first three images in row four represent the caudal midbrain; last two images in row four depict more rostral levels. Row five represents overlays of candidate genes (blue) with adjacent Th slide (red) from the same mouse. Schematic illustrations were adapted from Paxinos, G. & Franklin, K. B. J.^42^. Abbreviations: RLi, rostral linear nucleus; SNc, substantia nigra pars compacta; VTA, ventral tegmental area.

To compare gene expression profiles between the VTA and the SNc in the newborn mouse, the direct red fluorescence obtained from the reporter protein tdTomato (tdTom) was used to grossly outline the outer borders of the VTA and SNc which were dissected from *Dat-Cre*^*tdTom*^ pups at postnatal day 3 (P3) (Fig. 1a top). Whole cell RNA was prepared and analyzed for gene expression by the GeneChip^®^ Mouse Gene 1.0 ST Arrays; by using the entire cell material, a bias towards DA neurons was avoided which allowed composite gene expression analyses in all cell types. A total of 28270 genes were analyzed (Supplementary Table S1) and by plotting adjusted p-value over expression difference, 21 genes displayed at least log2-fold higher expression level in the VTA and 36 in the SNc (Fig. 1a and Supplementary Table S2). P-values were generally high so in order to sort the results, the top 50 genes according to p-value were chosen and thereafter sorted by log2-fold change (Fig. 1b bottom and c).

Based on a cut-off set at ±1.0 log2-fold change, the 15 highest ranked genes were selected for histological screening of mouse P3 midbrain sections by low-resolution *in situ* hybridization analysis using short ^35^S-labeled oligo-probes towards the mRNA (oligo sequences in Supplementary Table S3). These 15 genes were: *Gastrin-releasing peptide* (*Grp*), *Nicotinic acetylcholine receptor subunit alpha-2* (*Chrna2*), *Protein phosphatase-1 subunit-17* (*Gsbs*), *Transient receptor potential cation channel subtype V1* (*TrpV1*), *Melanocortin-3-receptor* (*Mc3r*), *Neuropilin-2* (*Nrp2*), *Cysteine-rich secretory protein-1* (*Crisp1*), *G-protein-coupled receptor-83* (*Gpr83*), *Neurotrophin-3* (*Ntf3*), *Tachykinin-receptor-3* (*Tacr3*), *Neuromedin-U* (*Nmu*) and *Follistatin (Fst)* which all showed higher expression in the VTA, and the *Zinc-finger-protein-of-the-cerebellum-2 (Zic2)*, *Serine peptidase inhibitor subtype-f1* (*Serpinf1*) and *Sine-oculis-related homeobox-3* (*Six3*) genes that were higher in the SNc samples. In addition to these 15 genes, *Calbindin1* (*Calb1)* and *Neuronal differentiation-6 (NeuroD6*) which showed low statistical ranking were included (Supplementary Table S1) as these were reported in previous microarray studies comparing gene expression profiles selectively between DA neurons of the VTA and SNc^6^,^7^.

The histological screening revealed that neither *Chrna2, Gsbs, Mc3r, Crisp1, Gpr83, Nmu, Zic2* nor *Serpinf1* were detected in either the VTA or the SNc at P3 (Fig. 1d and Supplementary Fig. S1) and were therefore excluded from further analysis. In contrast, *Six3* expression could be found in the SNc area while *Grp, TrpV1, Nrp2, Ntf3, Tacr3, Calb1 and NeuroD6* genes were absent from the SNc, but showed various expression patterns within the VTA: Grp mRNA was found in the ventromedial VTA; TrpV1 mRNA was even more medially restricted than Grp mRNA; Ntf3, Tacr3 and Calb1 mRNAs appeared more caudal and lateral than Grp, while Fst and NeuroD6 were similar but not identical to Grp mRNA within the VTA (Fig. 1d and Supplementary Fig. S1). The patterns of these 8 genes were of interest to map with anatomical detail and next, riboprobe high-resolution *in situ* hybridization was performed.

### Identification of *Calb1, Grp, Lipoprotein lipase (Lpl), NeuroD6* and *TrpV1* gene expressions as restricted to VTA subnuclei

**Figure 2:**
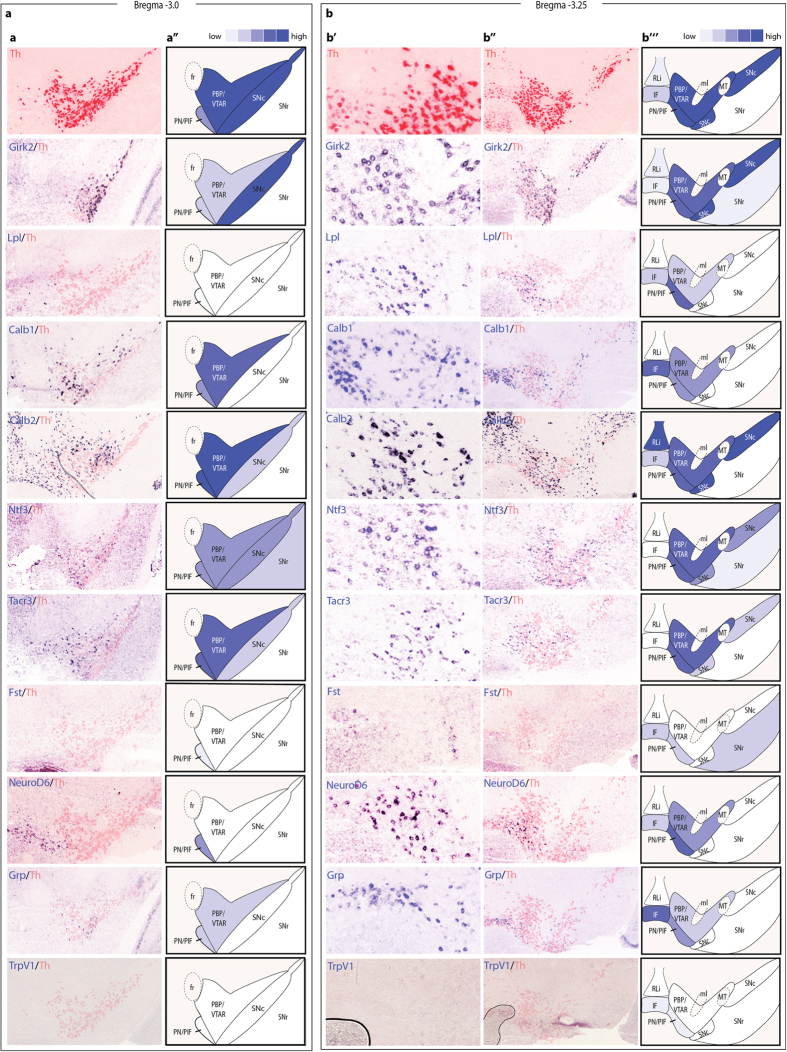
Identification of expression patterns in the VTA and SN of the adult mouse. (**a’**,**b’**,**b’’**) Riboprobe *in situ* hybridization analysis of candidate genes in adult C57BL/6 mice; row 2 onwards–expression pattern of adjacent Th slide in the same animal as red overlay (reduced intensity in red channel for visualization purposes) over expression pattern of the analyzed gene. All images were cropped from whole slides acquired with a digital high resolution slide scanner. (**a’’**,**b’’’**) Schematic visualization of expression patterns of the candidate genes. Schematic illustrations were adapted from Paxinos, G. & Franklin, K. B. J.^42^. Abbreviations: IF, interfascicular nucleus; ml, medial lemniscus; MT, medial terminal nucleus; PBP/VTAR, parabrachial nucleus + ventral tegmental area rostral part; PN/PIF, paranigral nucleus + parainterfascicular nucleus; RLi, rostral linear nucleus; SNc, substantia nigra pars compacta; SNr, substantia nigra pars reticulata.

Overlays of images from high-resolution *in situ* hybridization of the 8 mRNAs (Grp, TrpV1, Nrp2, Ntf3, Tacr3, Calb1 and NeuroD6) with adjacent sections showing Th mRNA, used as a reference, guided the mapping in the adult midbrain (Fig. 2) according to previous publications^44^,^45^,^46^. The *Six3* riboprobe appeared non-selective and was therefore excluded. All other expressions are shown in Fig. 2a,b and summarized in Table 1. The VTA was structured into the following subnuclei: the parabrachial pigmented nucleus (PBP) and VTA rostral nucleus (VTAR) (PBP/VTAR; corresponds to only PBP in studies prior to Fu *et al*.^44^); the paranigral nucleus (PN) and parainterfascicular nucleus (PIF) (PN/PIF; corresponds to only PN prior to Fu *et al*.^44^); the interfascicular nucleus (IF) and the rostral linear nucleus (RLi). The caudal linear nucleus (CLi) was not analyzed while the ventrally located supramammillary nucleus (SuM) was used as a ventral reference structure. Strong *Th* gene expression was as expected found in the lateral areas corresponding to PBP/VTAR, PN/PIF and SNc, while a weaker expression was observed in the medial IF and RLi and was absent as expected from the GABA-rich SN pars reticulata (SNr).

*Girk2* and *Lpl*, previously identified in microarray screens as elevated in the SNc over the VTA, and vice versa^6^,^7^,^8^, were included for histological validation. Girk2 mRNA appeared in all *Th*-expressing areas of the VTA and SNc, stronger laterally than medially, but not exclusive for SNc. Lpl mRNA was not detected in either the SNc or the adjacent SNr but was restricted to the VTA, and was strongest within the PN/PIF area. The *Calb2* gene was included for comparison with *Calb1*. In contrast to a previous study localizing protein distribution^16^, Calb1 and Calb2 mRNA showed almost opposite mapping patterns: Calb1 mRNA was most prominent in the medially located IF but was also detected within the laterally located PBP/VTAR and PN/PIF areas while Calb2 mRNA was strong in the RLi and lateral VTA as well as SNc and almost excluded from the IF. Similar to *Calb2*, the *Ntf3*, *Tacr3* and *Fst* genes were also excluded, or almost so, from the IF but were present at various degrees in the laterally located PBP/VTAR and PN/PIF; *Ntf3* was broadly expressed throughout the SNr and SNc, while Fst mRNA was very weakly detected in the SNr and Tacr3 mRNA in the SNc.

Three genes, namely *NeuroD6*, *Grp* and *TrpV1*, in addition to *Calb1 and Lpl* described above, showed expression exclusively within subnuclei of the VTA and were not detected at all in either SNr or SNc. Of these, NeuroD6 mRNA appeared as weak as Lpl mRNA in the IF but was detected in the PBP/VTAR. Both NeuroD6 and Lpl mRNAs were at their strongest in the PN/PIF area. An almost opposite pattern was seen with *Grp* which was expressed mostly within the IF and less in PN/PIF and PBP/VTAR areas. TrpV1 mRNA was detected in only few cells distributed within the IF and PN/PIF. Taken together, these histological analyses demonstrate several new expression patterns within the analyzed midbrain areas. Of these, five patterns are exclusive for subnuclei within the VTA: NeuroD6 and Lpl mRNAs are stronger in the lateral VTA while Calb1, TrpV1 and Grp mRNAs are stronger in the medial VTA of the adult mouse.

### *Calb1, Grp, NeuroD6, Ntf3* and *Tacr3* expression mainly in dopaminergic neurons

**Figure 3:**
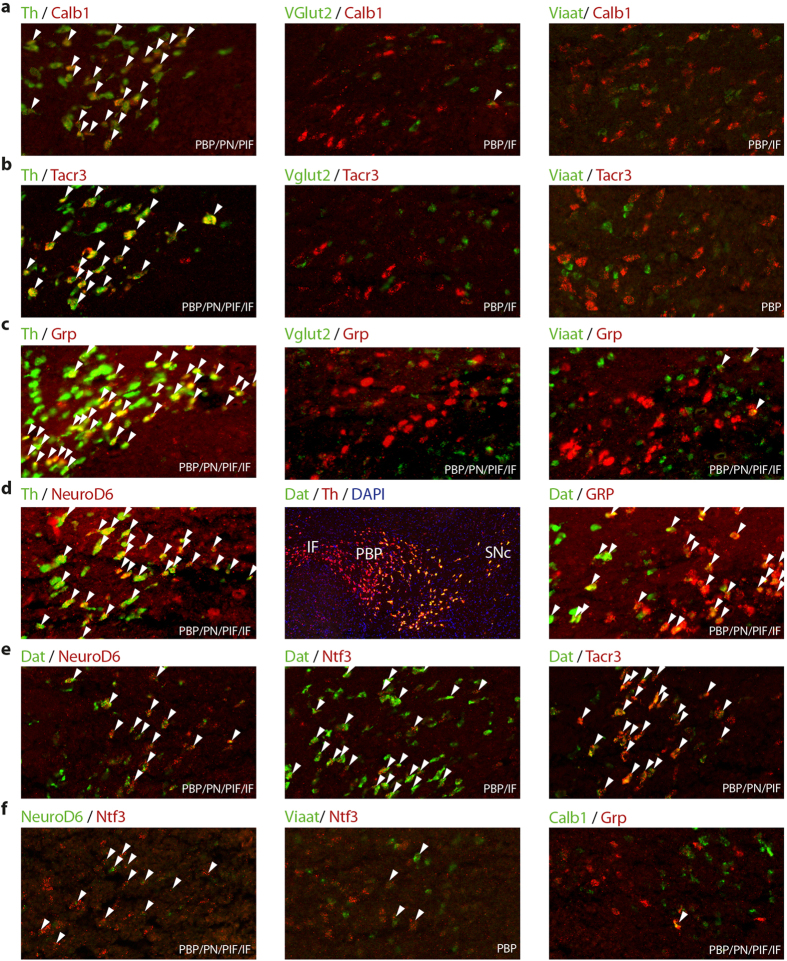
Double-fluorescence *in situ* hybridization reveals subpopulations of dopaminergic neurons in the VTA subnuclei. **(a–f)** Representative images depicting sdFISH results with fluorescein–labeled (green) and Digoxigenin-labeled (red) probes for different markers. White arrows indicate cell co-expressing both markers analyzed. All images were cropped from whole slides acquired with a digital high resolution slide scanner. Abbreviations: sdFISH, mRNA-selective double-fluorescent *in situ* hybridization; IF, interfascicular nucleus; PBP/VTAR, parabrachial nucleus + ventral tegmental area rostral part; PN/PIF, paranigral nucleus + parainterfascicular nucleus.

To validate the neurotransmitter identity of the VTA cells, mRNA-selective double-fluorescent *in situ* hybridization (sdFISH) analysis of Calb1, Grp, NeuroD6, Ntf3, Tacr3, and Trpv1 mRNAs was performed. Probes towards Th and Dat mRNAs were included for detection of dopaminergic neurons, Vglut2 mRNA for glutamatergic neurons and the Vesicular inhibitory amino acid transporter (Viaat) mRNA for inhibitory neurons. TrpV1 mRNA analysis gave no conclusive results due to the scarcity of positive cells, but Calb1 (Fig. 3a), Tacr3 (Fig. 3b), Grp (Fig. 3c), NeuroD6 (Fig. 3d,e) mRNA all co-localized with Th and/or Dat mRNA with no or very little co-localization with either Vglut2 or Viaat. Ntf3 mRNA co-localized mainly with Dat but some overlap with Viaat mRNA was also seen (Fig. 3e,f). Comparing Th and Dat mRNA confirmed previous reports^37^,^47^,^48^ of higher level of Dat mRNA within laterally distributed *Th*-expressing cells of the VTA and SNc (Fig. 3d). Accordingly, the laterally expressed *NeuroD6* gene co-localized entirely with both Th and Dat mRNA, Tacr3 and Ntf3 mRNA overlapped substantially with both, and the medially expressed *Grp* gene overlapped only partially with Dat mRNA (Fig. 3d,e). NeuroD6 and Ntf3 mRNA also showed some overlap with each other while Grp and Calb1 mRNA showed very little overlap (Fig. 3f). Overall, the *Grp, Calb1, Tacr3, Ntf3 and NeuroD6* genes were demonstrated as mainly expressed in DA cells, with only Nft3 mRNA as a putative marker for some GABA or GABA/DA co-releasing cells, while none of the mRNAs co-localized with Vglut2 mRNA.

### *TrpV1-Cre* cells in the VTA show low I_h_-currents and are mostly glutamatergic

**Figure 4:**
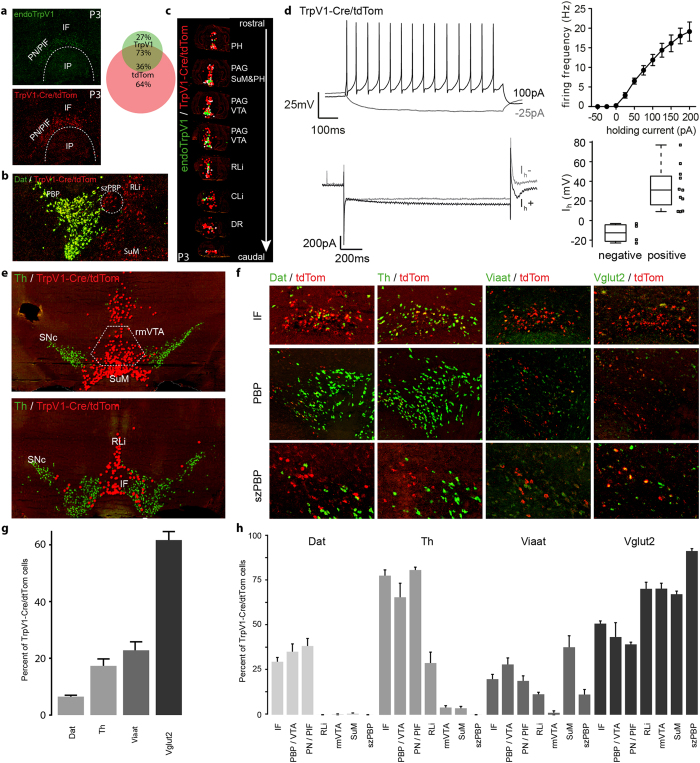
TrpV1-Cre cells in the VTA represent a mixed phenotype of glutamateric, dopaminergic and GABAergic neurons. **(a)** endoTrpV1 (top, green) and tdTom (bottom, red) sdFISH in *TrpV1-CretdTom* P3 mice. 73% of endoTrpV1 neurons are tdTom positive, 36% of tdTom cells contain endoTrpV1 at P3 (right). (**b)** Dat (green) and tdTom sdFISH. tdTom is restricted to the medial midbrain and labels a medial area in PBP low in dopaminergic neurons (szPBP). (**c)** Overview of endoTrpV1-only (green dots), tdTom-only (red dots) and co-expressing cells (white dots) from rostral to caudal midbrain. (**d)** Electrophysiological characterization of *TrpV1-cre*^*tdTom*^ cells in P10-18 *TrpV1-cre*^*tdTom*^ mice. Current-clamp recordings at different holding currents (top left); firing frequency during current injection (n = 10) (top right); Voltage-clamp recordings of I_h_ in I_h_- and I_h_+ cells (bottom left); Mean I_h_ at −132 mV holding potential in I_h_- (n = 4) and I_h_+ (n = 11) cells (bottom right). (**e)** Th (green) and tdTom sdFISH (red dots) in adult mice. tdTom labels a population of neurons rostral of the IF and caudal of the PH labeled rmVTA (top). (**f)** sdFISH of tdTom (red) with Dat (first column), Th (second column), Viaat (third column) and Vglut2 (fourth column) (green) in the IF (first row), PBP (second row) and szPBP (third row). (**g,h)** Manual cell-counting results of Dat-, Th-, Viaat and Vglut2-coexpression with tdTom in the VTA (G, subnuclei values in H) and SuM (H) in (n = 3) adult mice. All sdFISH images were cropped from whole slides acquired with a digital high resolution slide scanner. Abbreviations: CLi, caudal linear nucleus; DR, dorsal raphe nucleus; sdFISH, mRNA-selective double-fluorescent *in situ* hybridization; tdTom, tdTomato; endoTrpV1, endogenous TrpV1; IF, interfascicular nucleus; I_h_, hyperpolarization-activated current; PAG, periaqueductal grey; PH, posterior hypothalamus; PBP, parabrachial nucleus + ventral tegmental area rostral part; PN/PIF, paranigral nucleus + parainterfascicular nucleus; RLi, rostral linear nucleus; rmVTA, rostromedial ventral tegmental area; SNc, substantia nigra pars compacta; SuM, supramammillary nucleus; VTA, ventral tegmental area.

As *TrpV1* gene expression was strong in the medial VTA at P3 but only detected in few cells in the adult VTA, this gene was likely identified in the current microarray screen as a result of the approach taken; non-DA-biased and performed in the newborn mouse, as opposed to previous DA-selective approaches performed in adult mice, none of which reported *TrpV1*^6^,^7^. The TRPV1 molecule was originally identified as the capsaicin receptor and is strongly linked to endocannabinoid, heat and pain signaling^49^,^50^. Detection of TrpV1 mRNA within the VTA was therefore of particular interest and a *TrpV1*-Cre transgenic mouse tool^51^ was selected for further analyses of this discrete VTA-population. sdFISH analysis on midbrain sections derived from newborn *TrpV1-Cre*^*tdTom*^ mice showed that mRNA of the tdTom reporter was confined within the VTA while it was absent from the SNc, i.e. expression of the *TrpV1-Cre* transgene represented a similar spatial distribution pattern as the endogenous TrpV1 mRNA (Fig. 4a). Within the VTA, both endogenous TrpV1 and tdTom mRNAs were confined to scattered cells of the IF and PN/PIF areas, but tdTom mRNA was also detected within the RLi and in the medial aspect of the PBP/VTAR. Quantification revealed that 73% of all cells expressing the endogenous *TrpV1* gene in the VTA co-expressed the *tdTom*-reporter gene, while only 36% of the *tdTom*-expressing cells in the VTA area co-expressed the endogenous *TrpV1* gene (Fig. 4a).

Curiously, within the DA neuron-rich PBP area was a group of *TrpV1-Cre*-driven *tdTom-*expressing cells not co-expressing either *Th* or *Dat*, but that instead co-expressed the *Vglut2* gene; this particular PBP subarea was here given the name “subzone of the PBP” (szPBP) to distinguish its strongly glutamatergic nature from the surrounding DA cells (Fig. 4b,f).

Further, while endogenous TrpV1 mRNA within the midbrain was almost uniquely detected within the medially located VTA subnuclei (IF and PN/PIF), tdTom mRNA was not only broader within the VTA (IF, PN/PIF, RLi, medial PBP/VTAR) but was also detected medially within the dorsal aspect of the midbrain, confined to the periaqueductal grey (PAG). In addition, tdTom mRNA was found in a more rostral location than the endogenous TrpV1 mRNA, and was, as also described for a different *TrpV1-Cre* transgene^52^, detected in the posterior hypothalamus (PH) (Fig. 4c).

The *TrpV1-Cre* activity in the VTA was analyzed in further detail. Medial VTA neurons have been ascribed an electrophysiological profile of low hyperpolarization (I_h_) currents, as opposed to the higher currents displayed by lateral VTA neurons^27^. Basic electrophysiological properties of *TrpV1-Cre* positive cells in the VTA were therefore investigated by patch-clamp analyses in *TrpV1-Cre*^*tdTom*^ mice. Under current-clamp conditions, the patched *TrpV1-Cre*^*tdTom*^ cells, identified through direct red fluorescence, possessed a resting potential of −54.12 ± 2.75 mV and responded to stimulation with fast spiking activity up to 19.13 ± 2.44 Hz at 200 pA injected current (n = 10) (Fig. 4d). Whole-cell voltage-clamp experiments revealed that 73% of the patched cells (n = 11) were I_h_-positive with a low-I_h_ phenotype (32.88 ± 6.63 pA), while the remaining cells (n = 4) were I_h_-negative (Fig. 4d) and, thereby, the *TrpV1-Cre*^*tdTom*^ neurons represented an electrophysiological phenotype typical of the medial VTA.

Next, sdFISH protocols were implemented to pinpoint the extent of neurotransmitter cell types present in the heterogeneous VTA area that are represented by the *TrpV1-Cre* transgene. Co-expression of mRNA from the *TrpV1-Cre*-driven floxed reporter gene *tdTom* with mRNA for *Viaat* for GABA-signaling neurons, *Vglut2* for glutamatergic neurons and *Th* as well as *Dat* for dopaminergic neurons was analyzed (Fig. 4e–h).

Many *tdTom*-expressing cells were found in a location rostral of the IF and caudal of the PH, an area devoid of name in current atlases and indicated to contain only the retromamillary decussation^46^. This area, which similarly to the new szPBP was characterized by cells showing a high degree of co-localization of the *TrpV1-Cre*-driven tdTom mRNA and Vglut2 mRNA, is from here onwards referred to as the rostromedial VTA (rmVTA) and was included in the quantification (Fig. 4e).

Equally selective as endogenous TrpV1 mRNA for VTA subnuclei, sdFISH demonstrated a mixed neurotransmitter phenotype of the *TrpV1-Cre* cells (Fig. 4f). Quantification was performed throughout all VTA subnuclei in both newborn and adult *TrpV1-Cre* mice (Fig. 4g and Supplementary Table S5). Pooling all VTA subnuclei together, the majority (61.6 ± 3.0%) of the *TrpV1-Cre*-driven *tdTom*-cells in the adult mice co-expressed *Vglut2*, followed by co-expression with *Viaat* (22.8 ± 3.0%) *Th* (17.3 ± 2.4%) and *Dat* (6.5 ± 0.5%) (Fig. 4g) and similar results were found in the newborn mice (Supplementary Table S5). The lower co-expression with *Dat* than *Th* was in accordance with the more medial than lateral distribution of the *TrpV1-Cre*-driven *tdTom*-expressing cells within the VTA (Fig. 4f). When analyzing subnuclei separately, it was clear that the glutamatergic phenotype was prominent in the medial areas, including the IF, RLi, rmVTA and szPBP of the VTA as well as the SuM (Fig. 4h). Within the IF, PBP/VTAR and PN/PIF areas, the total amount of *Th*- and *Vglut2*-co-expressing *tdTom*-cells, respectively, added up to more than 100%, suggesting that some of these *TrpV1-Cre*-driven *tdTom*-cells thereby might be DA/glutamate co-releasing neurons. *TrpV1-Cre* expression therefore defines a mixture of glutamatergic, dopaminergic and GABA-signaling neurons within the VTA and possibly also represents various co-releasing neurons that co-exist within the VTA. The results further show that the majority of *TrpV1-Cre* cells are glutamatergic.

### *TrpV1-Cre* cells project exclusively to the NAc shell within the striatal complex and release glutamate upon optogenetic stimulation

**Figure 5:**
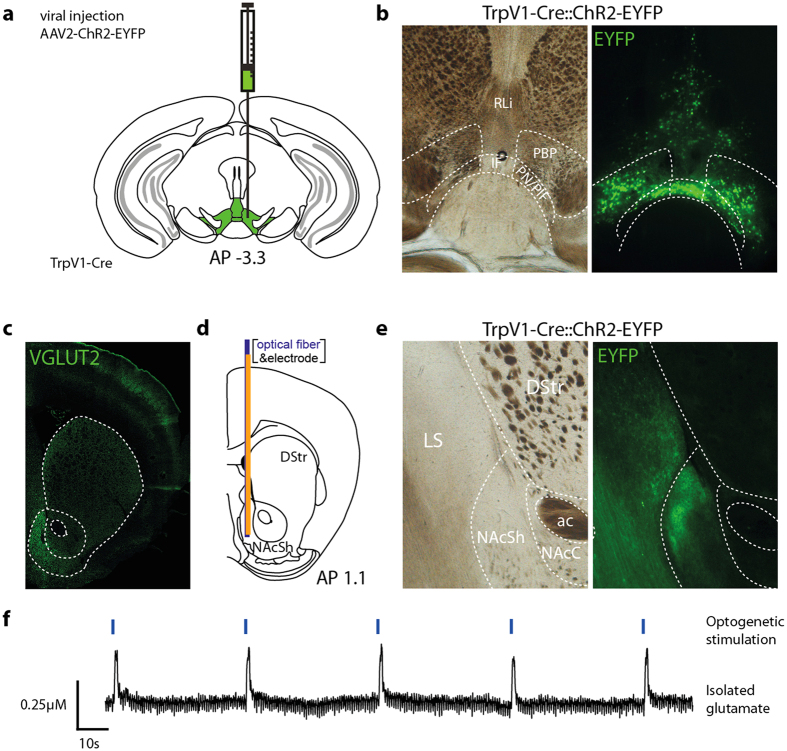
TrpV1-Cre positive projections release glutamate in the medial NAcSh upon optogenetic stimulation. **(a)** Schematic illustration of viral injection into the VTA at AP −3.3, ML −0.4 DV −4.7 & −4.2 mm in *TrpV1- Cre*^*tg/wt*^ mice (illustration kindly provided by Dr N. Schweizer). (**b)** Representative example of the injection area (left) and ChR2-EYFP expression (right) in TrpV1-Cre mice. (**c)** VGLUT2-immunofluorescent analysis in the recording area. (**d)** Schematic illustration of the recording complex [microelectrode (orange) and optical fiber (blue)] placement in the NAcSh. (**e)** Representative example of the recording area (left) and ChR2-expression (right) in TrpV1-Cre mice. (**f)** Representative example of five consecutive blue light stimulations (40 Hz, 1 s) at 4 mW (blue marks, top) and the isolated glutamate response (black trace, bottom). Image in (c) was cropped from whole slides acquired with a microscope slide scanner. Schematic illustrations were adapted from Paxinos, G. & Franklin, K. B. J.^42^. Abbreviations: ac: anterior commissure; DStr: dorsal striatum; EYFP: enhanced yellow fluorescent protein; IF, interfascicular nucleus; LS: lateral septum; NacC: nucleus accumbens core; NacSh: nucleus accumbens shell; PBP/VTAR, parabrachial nucleus + ventral tegmental area rostral part; PN/PIF, paranigral nucleus + parainterfascicular nucleus.

Based on the striking location of the *TrpV1-Cre* neurons exclusively within the VTA, their exclusion from the SNc and their strongly glutamatergic nature, it was of particular interest to functionally ascertain where these neurons project and if they there release measurable amounts of glutamate. To this end, a floxed AAV-ChR2-EYFP virus was injected into the VTA of *TrpV1-Cre* mice (Fig. 5a). Post-mortem histological analyses showed EYFP-positive cell bodies exclusively located within the medial part of the VTA of *TrpV1-Cre::ChR2-EYFP* mice (Fig. 5b). Thus, by implementing stereotactic virus injections, *TrpV1-Cre* transgenic mice can be used to selectively target the medial VTA neurons while leaving both lateral VTA and SNc structures as well as the dorsal PAG and rostral PH structures untargeted. In this way, the *TrpV1-Cre* transgene is spatially more restricted within the VTA than the *Th-Cre*, *Dat-Cre*, *Vglut-Cre* and *Gad-Cre* transgenic tools that have been used in previous optogenetic analyses of the VTA (reviewed in refs 17,42,43).

EYFP-positive fibers were sparse but possessed similar distributions as VGLUT2-positive terminals within the lateral septum and the NAc medial shell (NAcSh) while no EYFP fibers detected were in other parts of the NAc or the dorsal striatum which are target areas for dopaminergic neurons (Fig. 5c,e). To assess the functional properties of the newly identified pathway between the rostromedial VTA and the NAcSh, glutamate release was measured in the target area of *TrpV1-Cre::ChR2-EYFP* mice by *in vivo* amperometric recordings in which a glutamate-sensitive microelectrode was coupled in a complex with an optical fiber (Fig. 5d,e). Basal glutamate levels of 0.7 ± 0.3 μM were determined in the medial NAcSh. In control animals lacking ChR2-expression in the VTA and NAc, no detectable glutamate release was present upon optic stimulation. *TrpV1-Cre::*ChR2-EYFP expressing mice on the other hand exhibited reliable glutamate release of 0.10 ± 0.04 μM (n = 5 mice, average of five consecutive stimulations each) upon optical stimulations of 1 s at 40 Hz (Fig. 5f). In summary, by demonstrating that *TrpV1-Cre* cells of the rostromedial VTA project to the medial NAcSh region and there release glutamate upon activation, this study has identified the so far anatomically most restricted mesoaccumbal glutamatergic pathway.

### GRP and TRPV1 expression patterns identified in the human SNc

**Figure 6:**
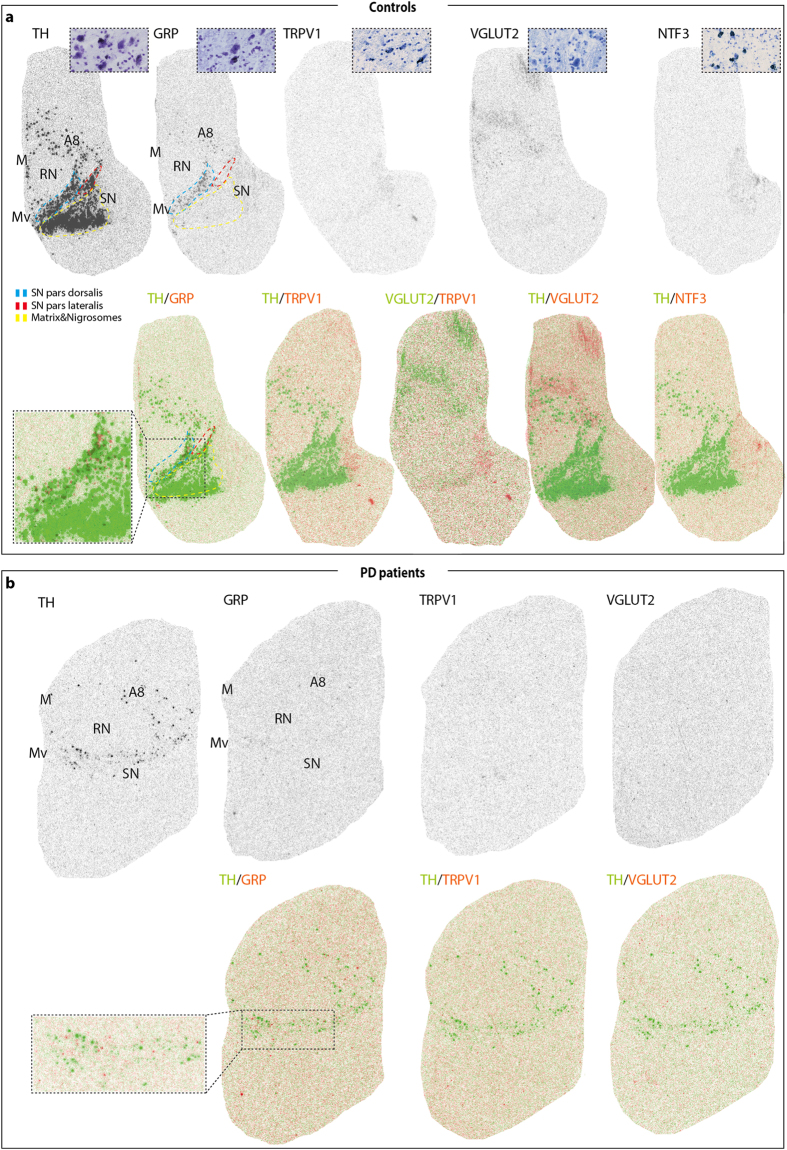
GRP and TRPV1 are expressed in subpopulations of substantia nigra neurons in the adult human brain and GRP remains in PD. **(a)** Overview of oligo-ISH of TH, GRP, TRPV1, VGLUT2 and NTF3 in the midbrain of postmortem tissue obtained from control subjects (top large images) with close-up images of sections treated with photoemulsion (inset in the top right of each slide, black grains correspond to mRNA expression). Subareas of the SN: SN pars dorsalis (blue), pars lateralis (red) and matrix and nigrosomes (yellow). Overlay of TH expression with adjacent GRP, TRPV1, VGLUT2, NTF3 section and overlay of VGLUT2 expression with adjacent TRPV1 section (bottom row, same sections as in the top). (**b)** Overview of oligo-ISH of TH, GRP, TRPV1 and VGLUT2 in postmortem tissue obtained from PD patients (top) and overlay of TH with adjacent GRP, TRPV1 and VGLUT2 (bottom). GRP expression is present in TH-containing areas in control subjects and remains in PD subjects (A and B bottom row inset). Abbreviations: A8, region containing A8 dopaminergic neurons; ISH, *in situ* hybidization; M, medial group; Mv, medioventral group; PD, Parkinson’s disease; RN, red nucleus; SN, substantia nigra.

Next, human tissue sections were analyzed using oligo *in situ* hybridization probes to human TRPV1, GRP, NTF3 alongside TH and VGLUT2. As expected, TH expression was found throughout the SN (subdivided into a matrix area surrounding the five nigrosomes; the Substantia nigra *pars dorsalis* (SNpd); Substantia nigra *pars lateralis* (SNpl)), A8 and the Medial (M) and Medioventral (Mv) areas, while no TH-expression was seen in the red nucleus (Fig. 6a top left). According to literature, the SNpd corresponds to the PBP in rodents, while Mv is similar to other parts of the VTA, including most prominently the IF and PN^53^,^54^,^55^. NTF3 mRNA was not detected at all in the human midbrain (Fig. 6a top right). In contrast, GRP expression was found mainly in areas corresponding to similar areas in the rodent: most expression was seen in the SNpd (mouse PBP) but also some in the A8 and Mv (Fig. 6a top middle left). TRPV1 and VGLUT2 showed similar expression patterns in the human midbrain and were both faintly detected in the matrix region and in A8 and Mv (mouse VTA) (Fig. 6a top middle right). Most overlap was seen between TH and GRP, possibly because these were the most strongly expressed genes (Fig. 6a bottom row). In the brains derived from PD patients, none or very little VGLUT2 or TRPV1 expression was detected (Fig. 6b, middle right and right). TH expression was considerately less detectable than in control brains and only remained in few cells (Fig. 6b, left). Strikingly, GRP expression was almost as prominent as in the control brains (Fig. 6b, middle left). This was curious as DA cells are the primary cell type to degenerate in PD, and for this reason, the GRP-expressing cells were analyzed in more detail.

### GRP is mostly expressed in non-melanized neurons and are spared in PD

**Figure 7:**
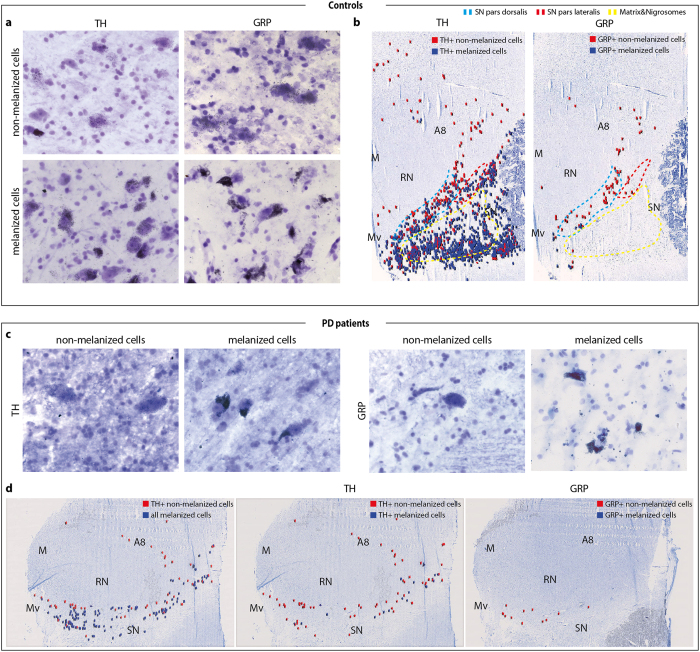
GRP is expressed in non-melanized cells in the human SN that are unaffected in PD. Sections treated with photoemulsion. (**a**,**b**) Postmortem tissue obtained from control subjects. (**a**) Representative example of non-melanized cells (first row) and melanized cells (second row) with TH (first column) and GRP expression (second column). (**b**) Overview of section with TH-positive (left) or GRP-positive (right) non-melanized (red) and melanized (blue) cells. Subareas of the SN: SN pars dorsalis (blue), pars lateralis (red) and matrix and nigrosomes (yellow). (**c,d**) Postmortem tissue obtained from PD patients. c: Representative example of TH (first two) and GRP expression (second two) in non-melanized cells (first and third) and melanized cells (second and fourth). (**d**) Overview of TH positive non-melanized cells (red) and all melanized cells (blue) (left); TH-positive (middle) or GRP-positive (right) non-melanized (red) and melanized (blue) cells. Abbreviations: A8, region containing A8 dopaminergic neurons; M, medial group; Mv, medioventral group; PD, Parkinson’s disease; RN, red nucleus; SN, substantia nigra.

The cause of the selective death of SNc over VTA DA neurons in PD is far from understood, but several studies have proposed the involvement of the pigment neuromelanin (NM) as DA neurons with high amounts of NM are more susceptible to degeneration^54^,^55^,^56^. High-resolution cellular analysis, in which the NM can be directly seen due to its dark color, revealed that GRP in the healthy brain is most strongly expressed within non-melanized TH neurons (Fig. 7a) which show a more restricted distribution than melanized TH cells. The non-melanized GRP-expressing neurons were localized within the SNpd and A8 while TH, which was detected within both melanized and non-melanized neurons, was seen throughout the SN, A8 and M, MV regions (Fig. 7b). Some weak GRP expression was, however, also detected within few melanized neurons in the Mv. In PD brains, melanized cells were mostly lost, and hence most of the TH-expression. The remaining TH expression was seen in non-melanized cells and the few remaining melanized neurons (Fig. 7c, left and d). In brain sections obtained from PD cases of different range of pathology, GRP expression was exclusively detected in non-melanized neurons in the PD brain. Further, with almost equally strong intensity between control and PD brains, this shows that non-melanized neurons expressing GRP are spared in PD (Fig. 7c right and d). Taken together, the histological analyses of human post-mortem brains demonstrate that while TRPV1 and VGLUT2 expression is low in both control and PD midbrain, the strong GRP expression remains in the PD midbrain: GRP can thereby be used as a new and selective marker for the non-melanized DA neurons that are neuroprotected in PD.

## Discussion

In contrast to the bias imposed towards DA neurons in previous expression analyses in rodents^6^,^7^,^8^,^9^,^10^,^16^, the current study implemented a crude mass-dissection approach of the VTA and SNc areas to obtain microarray-based gene expression data from all the different neuronal cell types that inhabit either the SNc or the VTA. Such gene expression patterns are essential in order to improve the current anatomical resolution in animal-based research centered around midbrain-disorders, but also to enhance the understanding of the etiology as well as to promote searches for new biomarkers of value for human therapies.

Summarizing the current findings, this study has shown that: (***i***) *Calb1*, *TrpV1* and *Grp* gene expression patterns represent medial VTA subnuclei while *NeuroD6* and *Lpl* represent lateral VTA subnuclei in the mouse; (***ii***) *Grp, Calb1, Tacr3, Ntf3 and NeuroD6* are mainly expressed in various DAergic subpopulations while *TrpV1-Cre*-expressing neurons in the VTA show a mixed neurotransmitter phenotype, representative of the heterogeneous VTA; (***iii***) Co-localization of *TrpV1-Cre*-expression with the glutamatergic Vglut2 mRNA marker enabled the identification of two new subnuclei in the medial VTA, i.e. the szPBP and rmVTA; (***iv***) Optogenetics in *TrpV1-Cre-*active VTA neurons identified a new glutamatergic mesoaccumbal pathway, a finding which makes this Cre-driver the most selective transgenic mouse tool for pre-clinical targeting within the VTA; (***v***) Human TRPV1 was sparse in the midbrain but GRP was readily detected in non-melanized DA neurons within the SN: GRP expression persisted in PD where melanized DA neurons were lost, a finding which demonstrates GRP as a marker for cells that are neuroprotected in PD.

By taking the finding of *TrpV1* expression within the rostromedial VTA at P3 through to functional transgenics- and optogenetics-based analyses in adult mice, the current study identified TrpV1 mRNA as spatially confined within the VTA and also exposed a group of VTA neurons, joined by their expression of the *TrpV1-Cre* transgene, with unique properties: This population of VTA neurons shows a restricted innervation pattern within the striatum exclusively to the NAcSh, a pattern which bears no resemblance in any other reported *Cre*-driver and further, although mainly glutamatergic, it is of mixed neurotransmitter phenotype, thus representing the heterogeneity of the VTA itself. Additionally, by characterizing the neurotransmitter identities of the *TrpV1-Cre* subpopulation within the VTA, it was possible to distinguish two novel subnuclei within the VTA that were characterized by *Vglut2*- and *TrpV1-Cre*-co-expression, the herein named szPBP and the rmVTA. While previous transgenics- and optogenetics-based studies have implemented neurotransmitter-selective Cre-drivers, including for example the broadly expressesd *Vglut2-Cre* transgene to successfully unravel target areas, signaling properties and behavioral roles of midbrain glutamatergic neurons^27^,^28^,^29^,^30^,^33^,^34^,^35^, implementation of anatomically restricted but heterogeneous Cre-drivers like *TrpV1-Cre* possess the spatial advantage of targeting neuronal populations that co-exist within subregions of the SNc and the VTA. The findings presented here strongly highlight the importance of identifying and mapping unique expression patterns and implementing these in functional assays for the value of increasing the anatomical detail in pre-clinical studies aimed dissecting out brain circuitries.

While GRP expression in the human midbrain was similar, but not identical, to that in the mouse, the situation was strikingly different for NTF3 with ample expression in the VTA of the mouse and no detectable expression at all in the human midbrain. Mice are often used to model human conditions, but this finding shows that there is no complete overlap in gene expression patterns between mouse and human midbrains, which is important to bear in mind when translating data between species. Post-mortem human material can, however, vary in terms of conservation which might cause differences in the limits of detection in histological assays, and based on ethical considerations, the use of human tissue was restricted here.

Previous histological studies have shown that medium-sized to large DA neurons express higher NM levels and are more susceptible to degeneration, and that within the VTA areas (SNpd, Mv), only half of the TH-positive population contains NM while the other half is non-melanized^54^,^55^,^56^,^57^. Curiously, in addition to enabling the use of GRP analysis for identification of those DA cells that are spared in PD, which might prove clinically useful, there is the possibility that GRP itself might be of therapeutic value as a neuroprotective factor. The implication of GRP in pathophysiology is far from new; both GRP analogues and GRP receptor antagonists are important targets in anti-cancer therapies already^58^,^59^ and both have been proposed as putative targets in neuropsychiatric disorders^60^,^61^,^62^. Indeed, GRP was listed as elevated in the VTA over the SNc in two separate “VTA *vs* SNc” screens over a decade ago^6^,^7^ and when PC12-cells over-expressing the *alpha-synuclein* gene were exposed to GRP, it was shown that these cultured cells were less vulnerable to the DA cell toxin MPP^+ ^6. Thus, almost 12 years after the identification of GRP as a neuroprotective factor in cell-based models of PD, the current study contributes with human mapping data that supports those previous findings.

Based on the findings presented here, by defining a restricted glutamatergic pathway between the VTA and NAcSh, TRPV1 might serve as an interesting target in future VTA-based studies, while the identification of GRP as a marker for non-melanized DA neurons that are neuroprotected in humans with PD could be of clinical value when exploring new therapies.

## Materials and Methods

### Ethics statement

All experimental protocols were in accordance with Swedish regulations, French Bioethical Laws and European Union legislation.

For all animal experiments, ethics approval was obtained from the Uppsala Animal Ethical Committee.

For analysis of human brain material, human brain tissue samples were obtained in a Brain Donation Program of the Brain Bank “GIE NeuroCEB” run by a consortium of Patients Associations: ARSEP (association for research on multiple sclerosis), CSC (cerebellar ataxias), France Alzheimer and France Parkinson. Informed consents were signed by the patients themselves or their next of kin in their name, in accordance with the French Bioethical Laws. The Brain Bank GIE NeuroCEB has been declared at the Ministry of Higher Education and Research and has received approval to distribute samples (agreement AC-2013–1887).

### Animals

*Dat-Cre*^62^ and *TrpV1-Cre*^47^ transgenic mice on mixed C57BL/6-129Sv background were bred to the red-fluorescent *Cre*-reporter mouse line *B6;129S6-Gt(ROSA)26Sor*^*tm9(CAG-tdTomato)Hze*^/J mice (Jax Mice^63^) to generate *Dat-Cre*^*tdTom*^(for microarray tissue preparation) and *TrpV1-Cre*^*tdTom*^(histological mapping, electrophysiology, optogenetics) mice in which the expression of *Cre recombinase* is driven by the transgenic *Dat* and *TrpV1* promoters. Genotyping for *Cre* and *tdTom* was performed on DNA extracted from ear biopsies using the following primer sequences: Cre FW ACGAGTGATGAGGTTCGCAAGA, Cre REV ACCGACGATGAAGCATGTTTAG; tdTom FW (transgene) CTGTTCCTGTACGGCATGG, tdTom REV (transgene) GGCATTAAAGCAGCGTATCC, tdTom FW (wildtype) AAGGGAGCTGCAGTGGAGTA, tdTom REV (wildtype) CCGAAAATCTGTGGGAAGTC.

### Tissue preparation and microarray analysis

Coronal brain slices (200μM thick) of P3 *DAT-Cre*^*tdTom*^mice (n = 4) obtained on a vibratome (Leica Microsystems, Germany) were left to recover in O_2_-saturated artificial CSF (oACSF) (125 mM NaCl, 25 mM NaHCO_3_, 2.5 mM KCl, 1.25 mM NaH_2_PO_4_, 2 mM CaCl_2_, 2 mM MgCl_2_, and 23 mM glucose). The SNc and VTA excluding the CLi and RLi were dissected and snap frozen on dry ice. Total RNA was extracted from individually dissected SN (n = 5) and VTA (n = 5) using the Qiagen RNeasy Mini Kit (Qiagen, Sweden). RNA expression was analyzed by microarray on GeneChip^®^ Mouse Gene 1.0 ST Arrays by the Uppsala Array Platform, Uppsala University as previously described^64^. The raw data was normalized with Expression Console (Affymetrix, USA) using the robust multi-array average (RMA) method^65^. Subsequent analysis of the gene expression data was carried out in R (http://www.r-project.org) using Bioconductor packages (www.bioconductor.org)^68^. An empirical Bayes moderated paired t-test was applied^66^ using the ‘limma’ package^67^ and p-values were adjusted^71^. Principal component analysis revealed that one of the 5 SN samples clustered further away than all other samples. This and the matching VTA sample were removed and the statistical analysis re-performed. The genes were ranked according to adjusted p-value and the 50 most significant genes ordered by log_2_-fold change between the SN and VTA.

### *In situ* hybridization on mouse and human tissue

Oligonucleotide and riboprobe sequences are listed in Supplementary Table S3. Radioactive *in situ* hybridization was performed on coronal cryosections derived from mouse and human brains as previously described^69^. For cellular mRNA expression analysis, slides were dipped in NTB emulsion, revealed after an exposition of 6 weeks and sections counterstained with toluidine blue. For non-radioactive *in situ* hybridization (colorimetric and fluorescent; sdFISH) analysis performed with riboprobes, cryosections were air-dried, fixed in 4% paraformaldehyde and acetylated in 0.25% acetic anhydride/100 mM triethanolamine (pH = 8). Sections were hybridized for 18 h at 65 °C in 100 μl of formamide-buffer containing 1 μg/ml digoxigenin (DIG) labelled probe for colorimetric detection or 1 μg/ml DIG and 1 μg/ml fluorescein-labelled probes for fluorescent detection. Sections were washed at 65 °C with SSC buffers of decreasing strength, and blocked with 20% FBS and 1% blocking solution. For colorimetric detection, DIG epitopes were detected with alkaline phosphatase-coupled anti-DIG fab fragments at 1/500 and developed with NBT/BCIP. For fluorescent detection, sections were incubated with HRP-conjugated anti-fluorescein antibody at 1/1000. Signal were revealed with the TSA™ Kit (Perkin Elmer) using Biotin-tyramide at 1:75 followed by incubation with Neutravidin Oregon Green conjugate at 1:750. HRP-activity was stopped by incubation of sections in 0,1 M Glycine and 3% H_2_O_2_. DIG epitopes were detected with HRP conjugated anti-DIG antibody at 1:1000 and revealed with TSA™ Kit (Perkin Elmer) using Cy3 tyramide at 1:200. All slides were scanned and analyzed on NanoZoomer *2.0-HT* Ndp2.view (Hamamatsu). The sdFISH method allows co-expression analysis of different mRNAs and thus enabled the use mRNA for the vesicular transporters, Viaat and Vglut2, to identify GABA- and glutamate-signaling neurons, respectively. As their protein products are localized in the synapses, only the use of mRNA-based methodology is useful for this purpose, while immunohistochemical approaches are not.

Midbrain sections at the SN level from control (n = 2; case number 3549, 8401) and PD patients (n = 3; case number 3392, 3490, 5874) (reference: GIE Neuro-CEB BB-0033-00011) were used. The pathology reports stated Diffuse Lewy body disease (DLB) for PD patient case number 3392 and 5874, and DLB + Alzheimer’s disease: Braak IV and Thal amyloid phase 1 + grain disease for PD case number 3490.

### Patch clamp electrophysiology

Coronal sections (300 μm) of P10-P18 *TrpV1-Cre*^*tdTom*^ mice were cut on a vibratome (Leica Microsystems, Germany) in sucrose-oACSF (2.5 mM KCl, 1.25 mM NaH_2_PO_4_, 26 mM NaHCO_3_, 10 mM D-glucose, 250 mM sucrose, 335 mOsmol) at 1–4 °C. The slices were kept in oACSF (126 mM NaCl, 2.5 mM KCl, 1.25 mM NaH_2_PO_4_, 26 mM NaHCO_3_, 10 mM D-glucose, osmolarity 290–300 mOsmol). During recordings, the slices were constantly perfused with 1.5 ml/min oACSF. Patch clamp experiments were performed using borosilicate glass electrodes (resistance 3–8 MΩ), filled with internal solution (0.13M K-gluconate, 7 mM NaCl, 0.1 mM EGTA, 10 mM HEPES, 0.3 mM Mg_2_Cl, 2 mM ATP, 0.5 mM GTP. pH 7.2–7.4, 290–300 mOsmol). Measurements were performed with Dagan BVC-700A and Dagan pc-one amplifiers (Dagan Corporation, USA), digitized with a National Instruments DAQ card and 2 kHz low-pass filtered (2x Bessel filter) signals acquired with winWCP (Dr. John Dempster, UK). Whole-cell voltage-clamp recordings were obtained for n = 15 *TrpV1*-positive cells (n = 7 mice) in the VTA (−60 mV to −132 mV in 10 steps of 8 mV). Whole-cell current-clamp recordings were carried out in n = 10 cells (n = 6 mice). (15 steps of 12.5 pA from −150 pA to +200 pA). Data analysis was performed using custom-written scripts in Matlab. For detection of evoked action potentials, the peak finder routine (Nathanael Yoder, http://www.mathworks.com/matlabcentral/fileexchange/25500-peakfinder-x0selthreshextremaincludeendpointsinterpolate) was used in combination with the custom scripts.

### Glutamate-selective *in vivo* amperometry upon optogenetic stimulation

*TrpV1-Cre* mice were anesthetized with isofluorane (0.5–2%) and stereotactic injections of optogenetics virus rAAV2-Ef1a-DIO-hChR2(H134R)-EYFP (courtesy of Dr. Karl Deisseroth’s lab, produced at UNC Vector Core) were performed at a concentration of ~3 × 10^12 at two dorsoventral (DV) levels at the following coordinates: −3.30 anteroposterior (AP) −0.40 mediolateral (ML) −4.2 and –4.7 DV (0.5 μl at each site) upon which the mice (*TrpV1-Cre::ChR2-EYFP*) recovered for three weeks. Microelectrodes with 4 recording sites (S2, Quanteon, USA) were coated with glutamate oxidase (2 sites) or BSA (2 sites) which allows for a self-referenced recording of glutamate release^70^. Microelectrodes were subsequently calibrated^72^, mounted with an optical fiber (200 μm diameter, Thorlabs, Inc., USA) and implanted in the NAcSh of *TrpV1-Cre::ChR2-EYFP* mice (n = 5; n = 3 lacking *ChR2-EYFP* expression and used as negative controls) using stereotaxic coordinates: AP +1.1, ML ± −0.52, and DV −4.0 mm under deep anesthesia (isofluorane with air (0,5–2 L/min, 1–3% v/v). Light stimulation was generated by a 473 nm MBL-III-473-100 mW laser (CNI Lasers, China) giving five subsequent light pulses, separated by 30 seconds at 40 Hz (5 ms pulses for 1 s, 4 mW output) whereupon glutamate release was recorded by the microelectrode maintained at a constant potential of +0.7 V. To control for possible light-induced artifacts, the protocol was repeated at a constant potential of +0.25 V and these amplitudes were subtracted from the amplitude at +0.7 V for each experiment. Raw data was analyzed using FAST analysis 6.1 (Jason Burmeister Consulting, USA) and custom R scripts (http://www.r-project.org). Basal glutamate levels were calculated and presented as difference between an enzyme-coated recording site and a non-coated sentinel site (subtracted signal)^72^.

## Additional Information

**How to cite this article**: Viereckel, T. *et al*. Midbrain Gene Screening Identifies a New Mesoaccumbal Glutamatergic Pathway and a Marker for Dopamine Cells Neuroprotected in Parkinson’s Disease. *Sci. Rep.* **6**, 35203; doi: 10.1038/srep35203 (2016).

## Supplementary Material

Supplementary Information

Supplementary Table S1

## Figures and Tables

**Table 1:** Summary of subnuclei mRNA pattern in the ventral midbrain of adult mice.

Adult	PBP + VTAR	PN + PIF	IF	RLi	SNc	SNr
Th	+++++	++++	++	+	+++++	−
Girk2	++++	++++	++	+	+++++	+
Calb1	++++	+++	++++	−	−	−
Calb2	+++++	+++++	+++	+++++	+++++	−
Lpl	+++	++++	++	−	−	−
NeuroD6	+++	++++	++	−	−	−
Grp	+++	++++	++++	−	−	−
Ntf3	+++	+++	−	−	−	+
Tacr3	++++	++++	−	−	++	−
Fst	−	++	++	−	−	++
TrpV1	−	+	+	−	−	−

Intensity according to an average of the schematics shown in Fig. 2a’’,b’’’ on a scale from no expression (white, **−**) to strong expression (dark blue, +++++) in a total of six levels (**−**, +, ++, +++, ++++, +++++). Abbreviations: IF, interfascicular nucleus; ml, terminal nucleus; PBP/VTAR, parabrachial nucleus + ventral tegmental area rostral part; PN/PIF, paranigral nucleus + parainterfascicular nucleus; RLi, rostral linear nucleus; SNc, substantia nigra pars compacta; SNr, substantia nigra pars reticulata.
