# Supplementary material for: Midbrain Gene Screening Identifies a New Mesoaccumbal Glutamatergic Pathway and a Marker for Dopamine Cells Neuroprotected in Parkinson’s Disease
Source: Sci Rep. 2016 Oct 20;6:35203. doi: 10.1038/srep35203 (PMC5071886; doi:10.1038/srep35203)
Supplement: Supplementary Information [file srep35203-s1.pdf]

Midbrain Gene Screening Identifies a New Mesoaccumbal Glutamatergic Pathway and a Marker for Dopamine Cells Neuroprotected in Parkinson's Disease

Thomas Viereckel[1,2], Sylvie Dumas[3,], Casey J.A. Smith-Anttila[1,4], Bianca Vclek[1], Zisis Bimpisidis[1], Malin C. Lagerström[2], Åsa Konradsson-Geuken[1], Åsa Wallén-Mackenzie[1*]

1. Department of Organismal Biology/Comparative Physiology, Uppsala University, S-752 36 Uppsala, Sweden

2. Department of Neuroscience, Uppsala University, S-751 24 Uppsala, Sweden.

3. Oramacell, 75006 Paris, France.

4. Department of Anatomy and Neuroscience, University of Melbourne, Victoria 3010, Australia

* Corresponding author: Professor Åsa Wallén-Mackenzie, Uppsala University, Department of Organismal Biology/Comparative Physiology, Norbyvägen 18A, S-752 36 Uppsala, Sweden. Telephone: +46-18-471 6419. E-mail: asa.mackenzie@ebc.uu.se

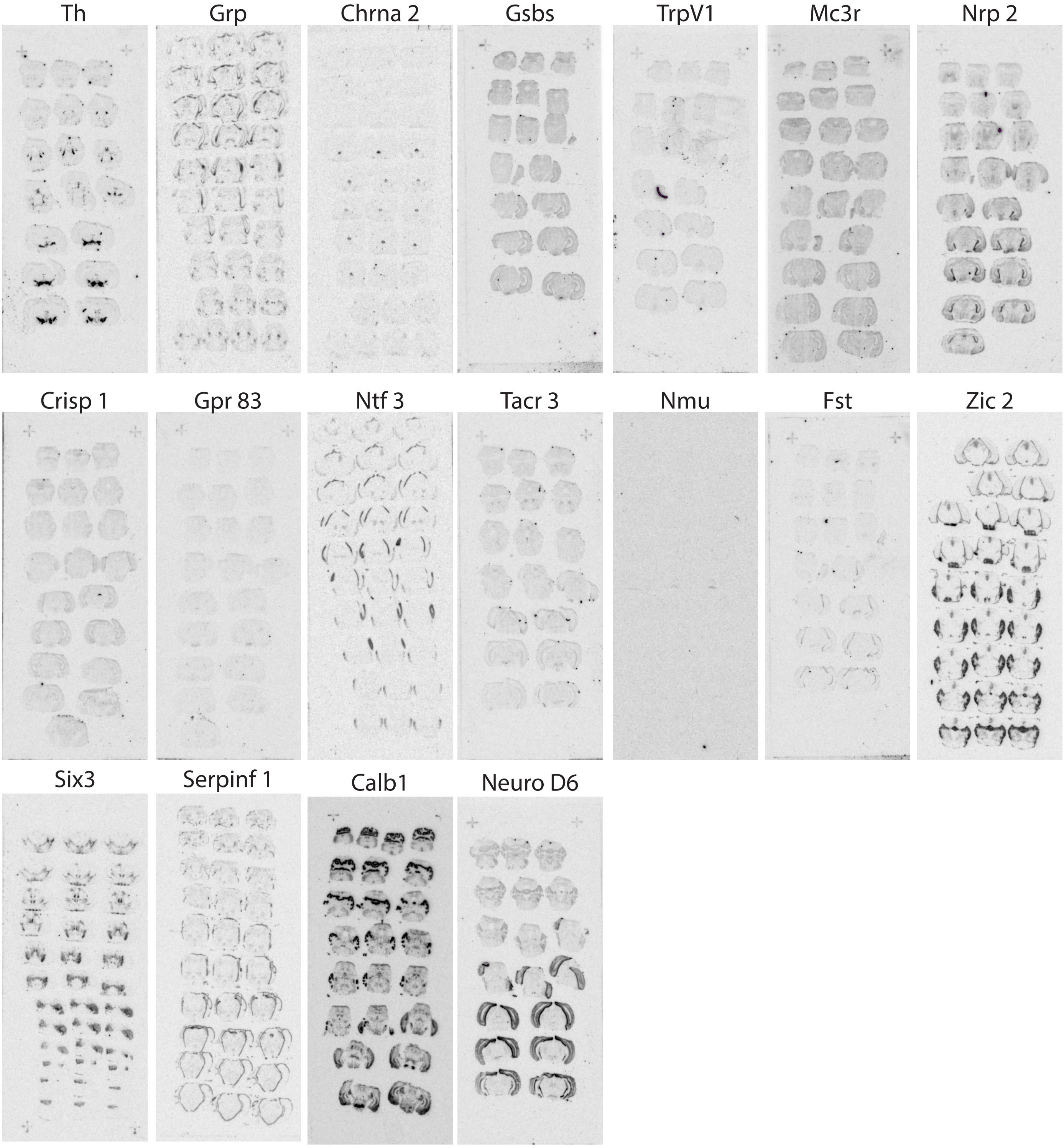

Supplementary Figure 1: Whole plate overview of oligo probe in situ hybridization results for each marker depicted in ***Figure 1D***.

Supplementary Table 2: GO categories for top 50 genes

Summary

Gene ontology term analysis by GO-Molecular Function revealed 16 of the candidate genes to be involved in transmembrane signaling transducer activity (p = 0.00016), 6 of which had G-protein coupled peptide receptor activity (p = 3.45e-05). When categorizing by GO-Biological Process, 17 genes were enriched in the category behavior (p = 1.53e-10) and 8 in axon guidance (p = 4.88e-06). A total of 15 genes were enriched in intrinsic component of the plasma membrane (p = 3.96e-05) and 14 in the synapse (p = 4.62e-08) by GO-Cellular Component.

Classification with Princeton Generic Gene GO Term Finder (Princeton University, USA; http://go.princeton.edu/cgi-bin/GOTermFinder) with mus musculus (MGI) as annotation background. Three separate queries were carried out for categorization according to molecular function (GO-MF), biological process (GO-BP) and cellular component (GO-CC).

Input list

Zic2, Six3, Serpinf1, Fign, Cdh12, Epha4, Ly96, Fkbp10, Accn4, Fbln1, Tmem90b, Gbx2, Clec1a, Adra1b, Lgi1, Mcc, Fam167a, Gpr52, Slit2, Csf2rb, Gabrq, Chrnb3, Sdc2, Scg2, Fgf10, Kcng3, Gch1, Ret, Necab1, C130021I20Rik, Kcnk9, Vwa5b1, Oprk1, Cckar, Cbln4, Lmx1a, Gpr98, Avpr1a, Fst, Nmu, Tacr3, Ntf3, Gpr83, Crisp1, Nrp2, Mc3r, Trpv1, Gsbs, Chrna2, Grp

Molecular function

Gene Ontology term	Cluster frequency	Genome frequency	Corrected P-value	FDR	false positives	Genes annotated to the term
G-protein coupled peptide receptor activity	6 of 50 genes, 12.0%	130 of 24183 genes, 0.5%	3.45E-05	0.00%	0	Mc3r, Oprk1, Cckar, Tacr3, Gpr83, Avpr1a
peptide receptor activity	6 of 50 genes, 12.0%	134 of 24183 genes, 0.6%	4.13E-05	0.00%	0	Mc3r, Oprk1, Cckar, Tacr3, Gpr83, Avpr1a
signaling receptor activity	17 of 50 genes, 34.0%	2082 of 24183 genes, 8.6%	5.63E-05	0.00%	0	Mc3r, Adra1b, Avpr1a, Nrp2, Epha4, Gabrq, Trpv1, Oprk1, Tacr3, Cckar, Gpr83, Chrna2, Ly96, Gpr98, Gpr52, Ret, Csf2rb
receptor activity	17 of 50 genes, 34.0%	2228 of 24183 genes, 9.2%	0.00014	0.00%	0	Mc3r, Adra1b, Avpr1a, Nrp2, Epha4, Gabrq, Trpv1, Oprk1, Tacr3, Cckar, Gpr83, Chrna2, Ly96, Gpr98, Gpr52, Ret, Csf2rb
transmembrane signaling receptor activity	16 of 50 genes, 32.0%	1988 of 24183 genes, 8.2%	0.00016	0.00%	0	Mc3r, Adra1b, Avpr1a, Nrp2, Epha4, Gabrq, Trpv1, Oprk1, Tacr3, Cckar, Gpr83, Chrna2, Gpr98, Gpr52, Ret, Csf2rb
signal transducer activity	17 of 50 genes, 34.0%	2280 of 24183 genes, 9.4%	0.0002	0.00%	0	Mc3r, Adra1b, Avpr1a, Nrp2, Epha4, Gabrq, Trpv1, Oprk1, Tacr3, Cckar, Gpr83, Chrna2, Ly96, Gpr98, Gpr52, Ret, Csf2rb
molecular transducer activity	17 of 50 genes, 34.0%	2425 of 24183 genes, 10.0%	0.00047	0.00%	0	Mc3r, Adra1b, Avpr1a, Nrp2, Epha4, Gabrq, Trpv1, Oprk1, Tacr3, Cckar, Gpr83, Chrna2, Ly96, Gpr98, Gpr52, Ret, Csf2rb
ion channel activity	7 of 50 genes, 14.0%	375 of 24183 genes, 1.6%	0.00141	0.00%	0	Gabrq, Trpv1, Kcnk9, Kcng3, Chrnb3, Accn4, Chrna2
extracellular ligand-gated ion channel activity	4 of 50 genes, 8.0%	69 of 24183 genes, 0.3%	0.00155	0.00%	0	Gabrq, Trpv1, Chrnb3, Chrna2
substrate-specific channel activity	7 of 50 genes, 14.0%	386 of 24183 genes, 1.6%	0.0017	0.00%	0	Gabrq, Trpv1, Kcnk9, Kcng3, Chrnb3, Accn4, Chrna2
calcium ion binding	8 of 50 genes, 16.0%	541 of 24183 genes, 2.2%	0.00172	0.00%	0	Gch1, Necab1, Slit2, Fkbp10, Fbln1, Gpr98, Cdh12, Ret
chemoattractant activity	3 of 50 genes, 6.0%	25 of 24183 genes, 0.1%	0.00227	0.00%	0	Scg2, Ntf3, Fgf10
channel activity	7 of 50 genes, 14.0%	409 of 24183 genes, 1.7%	0.00246	0.00%	0	Gabrq, Trpv1, Kcnk9, Kcng3, Chrnb3, Accn4, Chrna2
passive transmembrane transporter activity	7 of 50 genes, 14.0%	409 of 24183 genes, 1.7%	0.00246	0.00%	0	Gabrq, Trpv1, Kcnk9, Kcng3, Chrnb3, Accn4, Chrna2
cation channel activity	6 of 50 genes, 12.0%	278 of 24183 genes, 1.1%	0.00279	0.00%	0	Trpv1, Kcnk9, Kcng3, Chrnb3, Accn4, Chrna2
gated channel activity	6 of 50 genes, 12.0%	299 of 24183 genes, 1.2%	0.0042	0.12%	0.02	Gabrq, Trpv1, Kcnk9, Kcng3, Chrnb3, Chrna2

Biological Process

Gene Ontology term	Cluster frequency	Genome frequency	Corrected P-value	FDR	false positives	Genes annotated to the term
behavior	17 of 50 genes, 34.0%	794 of 24183 genes, 3.3%	1.53E-10	0.00%	0	Scg2, Mc3r, Ntf3, Fgf10, Adra1b, Six3, Avpr1a, Slit2, Epha4, Trpv1, Oprk1, Tacr3, Fign, Cckar, Gpr52, Serpinf1, Nmu
regulation of multicellular organismal process	22 of 50 genes, 44.0%	2614 of 24183 genes, 10.8%	1.88E-06	0.00%	0	Mc3r, Ntf3, Fgf10, Adra1b, Six3, Avpr1a, Slit2, Epha4, Trpv1, Oprk1, Mcc, Tacr3, Sdc2, Fst, Fbln1, Ly96, Serpinf1, Nmu, Tmem90b, Zic2, Ret, Lmx1a
locomotion	17 of 50 genes, 34.0%	1457 of 24183 genes, 6.0%	2.07E-06	0.00%	0	Scg2, Ntf3, Fgf10, Six3, Slit2, Nrp2, Gbx2, Epha4, Lgi1, Oprk1, Mcc, Cckar, Fbln1, Serpinf1, Zic2, Ret, Lmx1a
axon guidance	8 of 50 genes, 16.0%	193 of 24183 genes, 0.8%	4.88E-06	0.00%	0	Ntf3, Slit2, Gbx2, Nrp2, Epha4, Lgi1, Zic2, Lmx1a
neuron projection guidance	8 of 50 genes, 16.0%	194 of 24183 genes, 0.8%	5.08E-06	0.00%	0	Ntf3, Slit2, Gbx2, Nrp2, Epha4, Lgi1, Zic2, Lmx1a
cell morphogenesis involved in neuron differentiation	11 of 50 genes, 22.0%	512 of 24183 genes, 2.1%	5.19E-06	0.00%	0	Ntf3, Cckar, Slit2, Nrp2, Gbx2, Epha4, Lgi1, Sdc2, Zic2, Ret, Lmx1a
axonogenesis	10 of 50 genes, 20.0%	397 of 24183 genes, 1.6%	6.19E-06	0.00%	0	Ntf3, Cckar, Slit2, Nrp2, Gbx2, Epha4, Lgi1, Zic2, Ret, Lmx1a
neuron projection morphogenesis	11 of 50 genes, 22.0%	551 of 24183 genes, 2.3%	1.10E-05	0.00%	0	Ntf3, Cckar, Slit2, Nrp2, Gbx2, Epha4, Lgi1, Sdc2, Zic2, Ret, Lmx1a
axon development	10 of 50 genes, 20.0%	423 of 24183 genes, 1.7%	1.13E-05	0.00%	0	Ntf3, Cckar, Slit2, Nrp2, Gbx2, Epha4, Lgi1, Zic2, Ret, Lmx1a
cell morphogenesis involved in differentiation	12 of 50 genes, 24.0%	741 of 24183 genes, 3.1%	2.22E-05	0.00%	0	Ntf3, Cckar, Slit2, Nrp2, Gbx2, Epha4, Fbln1, Lgi1, Sdc2, Lmx1a, Zic2, Ret
chemotaxis	10 of 50 genes, 20.0%	484 of 24183 genes, 2.0%	4.01E-05	0.00%	0	Scg2, Ntf3, Fgf10, Slit2, Nrp2, Gbx2, Epha4, Lgi1, Zic2, Lmx1a
taxis	10 of 50 genes, 20.0%	486 of 24183 genes, 2.0%	4.17E-05	0.00%	0	Scg2, Ntf3, Fgf10, Slit2, Nrp2, Gbx2, Epha4, Lgi1, Zic2, Lmx1a
movement of cell or subcellular component	16 of 50 genes, 32.0%	1561 of 24183 genes, 6.5%	4.32E-05	0.00%	0	Scg2, Ntf3, Fgf10, Six3, Slit2, Nrp2, Gbx2, Epha4, Lgi1, Mcc, Cckar, Fbln1, Serpinf1, Zic2, Ret, Lmx1a
neuron development	13 of 50 genes, 26.0%	994 of 24183 genes, 4.1%	6.49E-05	0.00%	0	Ntf3, Cckar, Slit2, Nrp2, Gbx2, Epha4, Lgi1, Gpr98, Serpinf1, Sdc2, Lmx1a, Zic2, Ret
neuron projection development	12 of 50 genes, 24.0%	845 of 24183 genes, 3.5%	9.33E-05	0.00%	0	Ntf3, Cckar, Slit2, Nrp2, Gbx2, Epha4, Lgi1, Serpinf1, Sdc2, Lmx1a, Zic2, Ret
neuron differentiation	14 of 50 genes, 28.0%	1247 of 24183 genes, 5.2%	0.00012	0.00%	0	Ntf3, Cckar, Six3, Slit2, Nrp2, Gbx2, Epha4, Lgi1, Gpr98, Serpinf1, Sdc2, Lmx1a, Ret, Zic2
response to chemical	23 of 50 genes, 46.0%	3586 of 24183 genes, 14.8%	0.00012	0.00%	0	Gch1, Scg2, Ntf3, Fgf10, Adra1b, Avpr1a, Slit2, Nrp2, Gbx2, Epha4, Lgi1, Trpv1, Oprk1, Tacr3, Fst, Cckar, Gpr83, Ly96, Gpr98, Csf2rb, Zic2, Ret, Lmx1a
neurogenesis	15 of 50 genes, 30.0%	1474 of 24183 genes, 6.1%	0.00014	0.00%	0	Ntf3, Cckar, Fgf10, Six3, Slit2, Nrp2, Gbx2, Epha4, Lgi1, Gpr98, Serpinf1, Sdc2, Lmx1a, Ret, Zic2
regulation of cellular process	39 of 50 genes, 78.0%	10066 of 24183 genes, 41.6%	0.00014	0.00%	0	Mc3r, Ntf3, Adra1b, Avpr1a, Gbx2, Gabrq, Lgi1, Epha4, Oprk1, Kcng3, Tacr3, Necab1, Chrna2, Fbln1, Gpr52, Gsbs, Grp, Nmu, Lmx1a, Scg2, Gch1, Six3, Fgf10, Slit2, Nrp2, Trpv1, Mcc, Sdc2, Fign, Fst, Cckar, Gpr83, Ly96, Gpr98, Serpinf1, Csf2rb, Zic2, Tmem90b, Ret
response to stimulus	35 of 50 genes, 70.0%	8242 of 24183 genes, 34.1%	0.00019	0.00%	0	Mc3r, Ntf3, Adra1b, Avpr1a, Gbx2, Gabrq, Lgi1, Epha4, Oprk1, Tacr3, Chrna2, Fbln1, Gpr52, Grp, Nmu, Lmx1a, Scg2, Gch1, Six3, Fgf10, Slit2, Nrp2, Mcc, Trpv1, Fign, Fst, Cckar, Gpr83, Ly96, Gpr98, Serpinf1, Csf2rb, Zic2, Tmem90b, Ret
single organism signaling	29 of 50 genes, 58.0%	5799 of 24183 genes, 24.0%	0.00023	0.00%	0	Scg2, Mc3r, Ntf3, Fgf10, Adra1b, Six3, Avpr1a, Slit2, Nrp2, Gabrq, Epha4, Lgi1, Trpv1, Oprk1, Mcc, Tacr3, Fst, Cckar, Gpr83, Chrna2, Fbln1, Gpr98, Ly96, Gpr52, Grp, Nmu, Zic2, Csf2rb, Ret
signaling	29 of 50 genes, 58.0%	5807 of 24183 genes, 24.0%	0.00023	0.00%	0	Scg2, Mc3r, Ntf3, Fgf10, Adra1b, Six3, Avpr1a, Slit2, Nrp2, Gabrq, Epha4, Lgi1, Trpv1, Oprk1, Mcc, Tacr3, Fst, Cckar, Gpr83, Chrna2, Fbln1, Gpr98, Ly96, Gpr52, Grp, Nmu, Zic2, Csf2rb, Ret
signal transduction	28 of 50 genes, 56.0%	5439 of 24183 genes, 22.5%	0.00024	0.00%	0	Scg2, Mc3r, Ntf3, Fgf10, Adra1b, Six3, Avpr1a, Slit2, Nrp2, Gabrq, Epha4, Trpv1, Oprk1, Mcc, Tacr3, Fst, Cckar, Gpr83, Chrna2, Fbln1, Ly96, Gpr98, Gpr52, Grp, Nmu, Zic2, Csf2rb, Ret
regulation of signaling	19 of 50 genes, 38.0%	2557 of 24183 genes, 10.6%	0.00027	0.00%	0	Scg2, Ntf3, Fgf10, Adra1b, Six3, Avpr1a, Slit2, Epha4, Lgi1, Oprk1, Mcc, Fst, Cckar, Fbln1, Ly96, Gpr98, Nmu, Zic2, Ret
cell migration	13 of 50 genes, 26.0%	1128 of 24183 genes, 4.7%	0.00027	0.00%	0	Scg2, Ntf3, Cckar, Fgf10, Six3, Slit2, Nrp2, Gbx2, Epha4, Fbln1, Mcc, Serpinf1, Ret
regulation of cell communication	19 of 50 genes, 38.0%	2598 of 24183 genes, 10.7%	0.00035	0.00%	0	Scg2, Ntf3, Fgf10, Adra1b, Six3, Avpr1a, Slit2, Epha4, Lgi1, Oprk1, Mcc, Fst, Cckar, Fbln1, Ly96, Gpr98, Nmu, Zic2, Ret
cell communication	29 of 50 genes, 58.0%	5910 of 24183 genes, 24.4%	0.00035	0.00%	0	Scg2, Mc3r, Ntf3, Fgf10, Adra1b, Six3, Avpr1a, Slit2, Nrp2, Gabrq, Epha4, Lgi1, Trpv1, Oprk1, Mcc, Tacr3, Fst, Cckar, Gpr83, Chrna2, Fbln1, Gpr98, Ly96, Gpr52, Grp, Nmu, Zic2, Csf2rb, Ret
cell projection morphogenesis	11 of 50 genes, 22.0%	780 of 24183 genes, 3.2%	0.00036	0.00%	0	Ntf3, Cckar, Slit2, Nrp2, Gbx2, Epha4, Lgi1, Sdc2, Zic2, Ret, Lmx1a
generation of neurons	14 of 50 genes, 28.0%	1384 of 24183 genes, 5.7%	0.00043	0.00%	0	Ntf3, Cckar, Six3, Slit2, Nrp2, Gbx2, Epha4, Lgi1, Gpr98, Serpinf1, Sdc2, Lmx1a, Ret, Zic2
cell part morphogenesis	11 of 50 genes, 22.0%	801 of 24183 genes, 3.3%	0.00048	0.00%	0	Ntf3, Cckar, Slit2, Nrp2, Gbx2, Epha4, Lgi1, Sdc2, Zic2, Ret, Lmx1a
biological regulation	40 of 50 genes, 80.0%	11078 of 24183 genes, 45.8%	0.00063	0.00%	0	Mc3r, Ntf3, Adra1b, Avpr1a, Gbx2, Gabrq, Lgi1, Epha4, Oprk1, Kcng3, Tacr3, Necab1, Chrna2, Fbln1, Gpr52, Gsbs, Grp, Nmu, Lmx1a, Scg2, Gch1, Six3, Fgf10, Slit2, Nrp2, Trpv1, Mcc, Sdc2, Fign, Fst, Cckar, Gpr83, Ly96, Kcnk9, Gpr98, Serpinf1, Csf2rb, Zic2, Tmem90b, Ret
regulation of biological process	39 of 50 genes, 78.0%	10571 of 24183 genes, 43.7%	0.00067	0.00%	0	Mc3r, Ntf3, Adra1b, Avpr1a, Gbx2, Gabrq, Lgi1, Epha4, Oprk1, Kcng3, Tacr3, Necab1, Chrna2, Fbln1, Gpr52, Gsbs, Grp, Nmu, Lmx1a, Scg2, Gch1, Six3, Fgf10, Slit2, Nrp2, Trpv1, Mcc, Sdc2, Fign, Fst, Cckar, Gpr83, Ly96, Gpr98, Serpinf1, Csf2rb, Zic2, Tmem90b, Ret
cell motility	13 of 50 genes, 26.0%	1229 of 24183 genes, 5.1%	0.00073	0.00%	0	Scg2, Ntf3, Cckar, Fgf10, Six3, Slit2, Nrp2, Gbx2, Epha4, Fbln1, Mcc, Serpinf1, Ret
localization of cell	13 of 50 genes, 26.0%	1229 of 24183 genes, 5.1%	0.00073	0.00%	0	Scg2, Ntf3, Cckar, Fgf10, Six3, Slit2, Nrp2, Gbx2, Epha4, Fbln1, Mcc, Serpinf1, Ret
response to external stimulus	16 of 50 genes, 32.0%	1927 of 24183 genes, 8.0%	0.00076	0.00%	0	Scg2, Ntf3, Fgf10, Slit2, Nrp2, Gbx2, Epha4, Lgi1, Trpv1, Oprk1, Cckar, Ly96, Gpr98, Serpinf1, Zic2, Lmx1a

nervous system development	16 of 50 genes, 32.0%	1958 of 24183 genes, 8.1%	0.00094	0.00%	0	Ntf3, Fgf10, Six3, Slit2, Nrp2, Gbx2, Epha4, Lgi1, Sdc2, Cckar, Gpr98, Serpinf1, Tmem90b, Zic2, Ret, Lmx1a
cell projection organization	13 of 50 genes, 26.0%	1258 of 24183 genes, 5.2%	0.00095	0.00%	0	Ntf3, Cckar, Slit2, Nrp2, Gbx2, Epha4, Lgi1, Gpr98, Serpinf1, Sdc2, Lmx1a, Zic2, Ret
regulation of response to stimulus	20 of 50 genes, 40.0%	3085 of 24183 genes, 12.8%	0.00106	0.00%	0	Gch1, Scg2, Mc3r, Ntf3, Fgf10, Adra1b, Six3, Slit2, Epha4, Oprk1, Mcc, Tacr3, Fign, Fst, Fbln1, Ly96, Gpr98, Serpinf1, Zic2, Ret
positive regulation of multicellular organismal process	14 of 50 genes, 28.0%	1496 of 24183 genes, 6.2%	0.00109	0.00%	0	Fst, Ntf3, Fgf10, Adra1b, Avpr1a, Epha4, Trpv1, Ly96, Serpinf1, Tacr3, Nmu, Zic2, Tmem90b, Ret
digestive system process	5 of 50 genes, 10.0%	92 of 24183 genes, 0.4%	0.00112	0.00%	0	Trpv1, Oprk1, Cckar, Fgf10, Nmu
localization	26 of 50 genes, 52.0%	5192 of 24183 genes, 21.5%	0.00169	0.00%	0	Scg2, Chrnb3, Ntf3, Fgf10, Six3, Avpr1a, Slit2, Nrp2, Gbx2, Gabrq, Epha4, Trpv1, Oprk1, Kcng3, Mcc, Cckar, Accn4, Chrna2, Fbln1, Gpr98, Cbln4, Kcnk9, Serpinf1, Nmu, Tmem90b, Ret
cell development	16 of 50 genes, 32.0%	2059 of 24183 genes, 8.5%	0.00183	0.00%	0	Ntf3, Six3, Slit2, Nrp2, Gbx2, Epha4, Lgi1, Trpv1, Sdc2, Cckar, Fbln1, Gpr98, Serpinf1, Zic2, Ret, Lmx1a
central nervous system development	10 of 50 genes, 20.0%	737 of 24183 genes, 3.0%	0.00188	0.00%	0	Ntf3, Cckar, Fgf10, Six3, Slit2, Nrp2, Gbx2, Epha4, Zic2, Lmx1a
autonomic nervous system development	4 of 50 genes, 8.0%	45 of 24183 genes, 0.2%	0.00192	0.00%	0	Nrp2, Gbx2, Ntf3, Ret
cellular response to stimulus	30 of 50 genes, 60.0%	6845 of 24183 genes, 28.3%	0.00241	0.04%	0.02	Gch1, Scg2, Mc3r, Ntf3, Fgf10, Adra1b, Six3, Avpr1a, Slit2, Nrp2, Gabrq, Epha4, Trpv1, Oprk1, Mcc, Tacr3, Fign, Fst, Cckar, Gpr83, Chrna2, Fbln1, Gpr98, Ly96, Gpr52, Grp, Nmu, Zic2, Csf2rb, Ret
induction of positive chemotaxis	3 of 50 genes, 6.0%	14 of 24183 genes, 0.1%	0.00253	0.04%	0.02	Scg2, Ntf3, Fgf10
cell morphogenesis	12 of 50 genes, 24.0%	1181 of 24183 genes, 4.9%	0.0032	0.04%	0.02	Ntf3, Cckar, Slit2, Nrp2, Gbx2, Epha4, Fbln1, Lgi1, Sdc2, Lmx1a, Zic2, Ret
positive regulation of biological process	25 of 50 genes, 50.0%	5003 of 24183 genes, 20.7%	0.0033	0.04%	0.02	Gch1, Scg2, Mc3r, Ntf3, Fgf10, Adra1b, Six3, Avpr1a, Slit2, Gbx2, Lgi1, Epha4, Trpv1, Oprk1, Tacr3, Fst, Cckar, Fbln1, Gpr98, Ly96, Serpinf1, Nmu, Tmem90b, Zic2, Ret
ameboidal-type cell migration	7 of 50 genes, 14.0%	320 of 24183 genes, 1.3%	0.00347	0.04%	0.02	Nrp2, Gbx2, Mcc, Serpinf1, Fgf10, Ret, Slit2
digestion	5 of 50 genes, 10.0%	117 of 24183 genes, 0.5%	0.00368	0.04%	0.02	Trpv1, Oprk1, Cckar, Fgf10, Nmu
regulation of system process	8 of 50 genes, 16.0%	463 of 24183 genes, 1.9%	0.00384	0.04%	0.02	Mc3r, Fgf10, Adra1b, Avpr1a, Oprk1, Trpv1, Tacr3, Nmu
feeding behavior	5 of 50 genes, 10.0%	120 of 24183 genes, 0.5%	0.00417	0.04%	0.02	Mc3r, Oprk1, Cckar, Tacr3, Nmu
forebrain development	7 of 50 genes, 14.0%	338 of 24183 genes, 1.4%	0.00497	0.04%	0.02	Nrp2, Gbx2, Cckar, Fgf10, Six3, Lmx1a, Slit2
gastric acid secretion	3 of 50 genes, 6.0%	18 of 24183 genes, 0.1%	0.00565	0.04%	0.02	Trpv1, Cckar, Nmu
negative regulation of cell differentiation	9 of 50 genes, 18.0%	652 of 24183 genes, 2.7%	0.00566	0.04%	0.02	Fst, Fgf10, Six3, Slit2, Epha4, Fbln1, Gpr98, Trpv1, Lmx1a
cellular component morphogenesis	12 of 50 genes, 24.0%	1257 of 24183 genes, 5.2%	0.00607	0.04%	0.02	Ntf3, Cckar, Slit2, Nrp2, Gbx2, Epha4, Fbln1, Lgi1, Sdc2, Lmx1a, Zic2, Ret
tissue morphogenesis	9 of 50 genes, 18.0%	660 of 24183 genes, 2.7%	0.00624	0.04%	0.02	Fst, Fgf10, Six3, Slit2, Gbx2, Nrp2, Epha4, Zic2, Ret
anatomical structure morphogenesis	17 of 50 genes, 34.0%	2550 of 24183 genes, 10.5%	0.00644	0.03%	0.02	Scg2, Ntf3, Fgf10, Six3, Slit2, Nrp2, Gbx2, Epha4, Lgi1, Sdc2, Fst, Cckar, Fbln1, Serpinf1, Zic2, Ret, Lmx1a
locomotory behavior	6 of 50 genes, 12.0%	230 of 24183 genes, 1.0%	0.00661	0.03%	0.02	Epha4, Mc3r, Oprk1, Fign, Gpr52, Adra1b
negative regulation of developmental process	10 of 50 genes, 20.0%	863 of 24183 genes, 3.6%	0.00761	0.03%	0.02	Fst, Fgf10, Six3, Slit2, Epha4, Fbln1, Gpr98, Trpv1, Serpinf1, Lmx1a
diencephalon development	4 of 50 genes, 8.0%	65 of 24183 genes, 0.3%	0.00849	0.03%	0.02	Nrp2, Gbx2, Fgf10, Six3

Cellular Component

Gene Ontology term	Cluster frequency	Genome frequency	Corrected P-value	FDR	false positives	Genes annotated to the term
synapse	14 of 50 genes, 28.0%	792 of 24183 genes, 3.3%	4.62E-08	0.00%	0	Chrnb3, Cckar, Chrna2, Epha4, Lgi1, Gabrq, Trpv1, Gpr98, Cbln4, Kcnk9, Oprk1, Sdc2, Nmu, Tmem90b
intrinsic component of plasma membrane	15 of 50 genes, 30.0%	1577 of 24183 genes, 6.5%	3.96E-05	0.00%	0	Chrnb3, Cckar, Gpr83, Adra1b, Chrna2, Avpr1a, Epha4, Gabrq, Trpv1, Oprk1, Kcnk9, Ly96, Kcng3, Tmem90b, Ret
neuron part	14 of 50 genes, 28.0%	1376 of 24183 genes, 5.7%	4.78E-05	0.00%	0	Chrnb3, Cckar, Nrp2, Epha4, Trpv1, Gpr98, Kcnk9, Oprk1, Serpinf1, Tacr3, Sdc2, Nmu, Tmem90b, Ret
synapse part	9 of 50 genes, 18.0%	593 of 24183 genes, 2.5%	0.00031	0.00%	0	Chrnb3, Cckar, Chrna2, Epha4, Gabrq, Kcnk9, Trpv1, Tmem90b, Nmu
plasma membrane	24 of 50 genes, 48.0%	4662 of 24183 genes, 19.3%	0.00041	0.00%	0	Mc3r, Chrnb3, Fgf10, Adra1b, Avpr1a, Slit2, Nrp2, Gabrq, Epha4, Trpv1, Oprk1, Kcng3, Mcc, Cdh12, Tacr3, Cckar, Gpr83, Chrna2, Ly96, Kcnk9, Gpr98, Gpr52, Tmem90b, Ret
postsynaptic membrane	6 of 50 genes, 12.0%	214 of 24183 genes, 0.9%	0.00051	0.00%	0	Gabrq, Epha4, Trpv1, Chrnb3, Chrna2, Tmem90b
cell periphery	24 of 50 genes, 48.0%	4774 of 24183 genes, 19.7%	0.00064	0.00%	0	Mc3r, Chrnb3, Fgf10, Adra1b, Avpr1a, Slit2, Nrp2, Gabrq, Epha4, Trpv1, Oprk1, Kcng3, Mcc, Cdh12, Tacr3, Cckar, Gpr83, Chrna2, Ly96, Kcnk9, Gpr98, Gpr52, Tmem90b, Ret
integral component of plasma membrane	13 of 50 genes, 26.0%	1520 of 24183 genes, 6.3%	0.0009	0.00%	0	Chrnb3, Cckar, Gpr83, Adra1b, Chrna2, Avpr1a, Epha4, Gabrq, Oprk1, Kcng3, Kcnk9, Tmem90b, Ret
plasma membrane part	17 of 50 genes, 34.0%	2607 of 24183 genes, 10.8%	0.00102	0.00%	0	Chrnb3, Adra1b, Avpr1a, Epha4, Gabrq, Trpv1, Oprk1, Kcng3, Tacr3, Cckar, Gpr83, Chrna2, Ly96, Kcnk9, Gpr98, Ret, Tmem90b
neuron projection	11 of 50 genes, 22.0%	1084 of 24183 genes, 4.5%	0.00105	0.00%	0	Chrnb3, Cckar, Nrp2, Epha4, Trpv1, Oprk1, Serpinf1, Tacr3, Tmem90b, Ret, Nmu
synaptic membrane	6 of 50 genes, 12.0%	264 of 24183 genes, 1.1%	0.00171	0.00%	0	Gabrq, Epha4, Trpv1, Chrnb3, Chrna2, Tmem90b
integral component of membrane	25 of 50 genes, 50.0%	5625 of 24183 genes, 23.3%	0.00341	0.00%	0	Mc3r, Chrnb3, Clec1a, Adra1b, Avpr1a, Nrp2, Gabrq, Epha4, Trpv1, Oprk1, Kcng3, Cdh12, Tacr3, Sdc2, Cckar, Accn4, Gpr83, Chrna2, Gpr98, Ly96, Kcnk9, Gpr52, Tmem90b, Csf2rb, Ret
receptor complex	6 of 50 genes, 12.0%	307 of 24183 genes, 1.3%	0.00399	0.00%	0	Gabrq, Gpr98, Chrnb3, Ly96, Chrna2, Ret
intrinsic component of membrane	25 of 50 genes, 50.0%	5757 of 24183 genes, 23.8%	0.00516	0.00%	0	Mc3r, Chrnb3, Clec1a, Adra1b, Avpr1a, Nrp2, Gabrq, Epha4, Trpv1, Oprk1, Kcng3, Cdh12, Tacr3, Sdc2, Cckar, Accn4, Gpr83, Chrna2, Gpr98, Ly96, Kcnk9, Gpr52, Tmem90b, Csf2rb, Ret
cell body	8 of 50 genes, 16.0%	653 of 24183 genes, 2.7%	0.00539	0.00%	0	Epha4, Oprk1, Trpv1, Serpinf1, Tacr3, Sdc2, Tmem90b, Ret
axon	7 of 50 genes, 14.0%	487 of 24183 genes, 2.0%	0.00613	0.00%	0	Nrp2, Epha4, Oprk1, Cckar, Serpinf1, Nmu, Ret

Supplementary Table 3: Probe sequences for *in situ* hybridization

Mouse probes

mRNA	oligo name	NM	oligo seq	riboprobe seq
Calb1	Calb1-AS1	NM_009788.4	1508-1541	79-870
	Calb1-AS2		1362-1395	
	Calb1-AS3		860-893	
Lpl	Lpl-AS1	NM_008509.2	651-684	680-1460
	Lpl-AS2		1433-1466	
	Lpl-AS3		2954-2987	
Fst	Fst-AS1	NM_001301373.1	1586-1619	460-1221
	Fst-AS2		402-435	
	Fst-AS3		1875-1908	
NeuroD6	NeuroD6-AS1	NM_009717.2	99-132	635-1419
	NeuroD6-AS2		933-966	
	NeuroD6-AS3		1256-1288	
Tacr3	Tacr3-AS1	NM_021382.6	675-707	621-1427
	Tacr3-AS2		1052-1085	
	Tacr3-AS3		137-169	
Grp	Grp-AS1	NM_175012.3	86-119	134-858
	Grp-AS2		434-467	
	Grp-AS3		217-250	
Ntf3	Ntf3-AS1	NM_001164034.1	661-694	538-1295
	Ntf3-AS2		707-740	
	Ntf3-AS3		826-859	
Six3	six3-AS1	NM_011381.4	1400-1433	220-617
	six3-AS2		2521-2556	
	six3-AS3		389-422	
TrpV1	trpv1-AS1	NM_001001445.2	1441-1474	426-1239
	trpv1-AS2		2643-2676	
	trpv1-AS3		2102-2135	
Th	th-AS1	NM_009377.1	774-807	456-1453
	th-AS2		272-305	
	th-AS3		1621-1655	
tdTomato				84-653
slc17a6		NM_080853.3		2315-3244
slc32a1		NM_009508.2		649-1488
slc6a3		NM_012694.2		1015-1938
Girk2		NM_010606.2		275-1028
Calb1		NM_007586.1		80-809

mRNA	oligo name	NM	oligo seq
Crisp1	Crisp1-AS1	NM_009638.3	598-630
Gsbs	Ppp1r17-AS1	NM_011153.3	933-966
	Ppp1r17-AS2		159-192
	Ppp1r17-AS3		1602-1635
Mc3r	Mc3r-AS1	NM_008561.3	17-50
	Mc3r-AS2		108-141
	Mc3r-AS3		1963-1996
Gpr83	Gpr83-AS1	NM_010287.3	103-136
	Gpr83-AS2		1496-1529
Nrp2	Nrp2-AS1	NM_001077403.1	1773-1806
	Nrp2-AS2		1026-1059
	Nrp2-AS3		2349-2382
Chrna2	Chrna2-AS1	NM_144803.2	2316-2349
	Chrna2-AS2		3341-3375
	Chrna2-AS3		3031-3064
Nmu	Nmu-AS1	NM_019515.1	326-359
	Nmu-AS2		20-53
	Nmu-AS3		471-504
Serpinf1	Serpinf1-AS1	NM_011340.3	318-351
	Serpinf1-AS2		1269-1302
	Serpinf1-AS3		691-724
Zic2	Zic2-AS1	NM_009574.3	1835-1868
	Zic2-AS2		159-193
	Zic2-AS3		2291-2324

Supplementary Table 4: Summary of mRNA expression pattern in the ventral midbrain of P3 mice

P3	VTA	SNc	SNr
Th	+	+	-
Grp	+	-	-
Chrna2	-	-	-
Gsbs	-	-	-
TrpV1	+	-	-
Mc3r	-	-	-
Nrp2	+	-	-
Chrisp1	-	-	-
Gpr83	-	-	-
Ntf3	+	-	-
Tacr3	+	-	-
Nmu	-	-	-
Fst	+	-	-
Zic2	-	-	-
Six3	-	+	+
Serpinf1	-	-	-
Calb1	+	-	-
NeuroD6	+	-	-

Supplementary Table 5: Quantification of co-expression in P3 and adult mice

Quantification of Tomato and TrpV1, Th or Vglut2 co-expression in P3 mice

TrpV1	absolute cell counts			% coexpressing			
area	Tomato only	TrpV1 only	coexpressing	of tomato	of TrpV1	Tomato only (%)	TrpV1 only (%)
szPBP	10	0	5	33	100	67	0
rmVTA	28.5	12	15.5	35	58	42	65
rmVTA	33	6	19	37	76	24	63
RLI	31	9	17	36	67	33	64
RLI	32	8	18	36	71	29	64
PBP	32	8	18	36	71	29	64
PBP	32	8	18	36	71	29	64
IFC PN	32	8	18	36	71	29	64
IFC PN	32	8	18	36	71	29	64

Th	absolute cell counts			% coexpressing			
Area	Tomato only	Th only	coexpressing	of tomato	of Th	Tomato only (%)	Th only (%)
PBP	12	974	11	48	1	52	99
PN/PIF	25	352	100	80	22	20	78
RLi	63	21	4	6	16	94	84
rmVTA	83	34	6	7	15	93	85
szPBP	22	30	0	0	0	100	100

Vglut2	absolute cell counts			% coexpressing			
Area	Tomato only	Vglut2 only	coexpressing	of tomato	of Vglut2	Tomato only (%)	Vglut2 only (%)
PBP	23	93	10	30	10	70	90
PN/PIF	101	16	9	8	36	92	64
RLi	11	103	50	82	33	18	67
rmVTA	13	88	29	69	25	31	75
szPBP	2	24	11	85	31	15	69

Quantification of Tomato and Th, vglut2, viaat or Dat co-expression in adult mice

Dat				absolute cell counts		% coexpressing	
animal	area	section	Tomato only	Dat only	coexpressing	of tomato	of Dat
35402	IF	S139	14	10	5	26.32	33.33
35402	IF	S151	15	9	6	28.57	40.00
47367	IF	S102	13	11	7	35.00	38.89
47367	IF	S114	10	5	3	23.08	37.50
47319	IF	S111	13	10	7	35.00	41.18
35402	PBP	S139	11	242	4	26.67	1.63
47367	PBP	S114	17	195	7	29.17	3.47
47369	PBP	S111	11	204	9	45.00	4.23
47369	PBP	S123	12	NC	8	40.00	NC
47369	szPBP	S111	3	8	0	0.00	0.00
47369	szPBP	S123	14	25	0	0.00	0.00
47367	szPBP	S114	10	6	0	0.00	0.00
35402	szPBP	S151	20	20	0	0.00	0.00
35402	PN/PIF	S139	16	38	7	30.43	15.56
47367	PN/PIF	S114	21	82	17	44.74	17.17
47319	PN/PIF	S123	6	46	4	40.00	8.00
47367	rmVTA	S150	113	0	0	0.00	0.00
35402	rmVTA	S170	154	0	1	0.65	0.00
47319	rmVTA	S147	93	3	0	0.00	0.00
35402	SuM	S170	154	0	1	0.65	0.00
47319	SuM	S147	170	3	2	1.16	40.00
47367	SuM	S150	217	0	0	0.00	0.00
35402	RLi	S139	28	0	0	0.00	0.00
47367	RLi	S114	31	0	0	0.00	0.00

Th				absolute cell counts		% coexpressing	
animal	area	section	Tomato only	Th only	coexpressing	of tomato	of Th
35402	IF	S133	3	16	9	75.00	36.00
35402	IF	S145	4	16	11	73.33	40.74
47367	IF	S97	2	32	13	86.67	28.89
47367	IF	S109	3	34	16	84.21	32.00
47319	IF	S109	6	20	14	70.00	41.18
35402	PBP	S145	2	211	2	50.00	0.94

47367	PBP	S109	9	224	23	71.88	9.31
47369	PBP	S121	3	198	9	75.00	4.35
47369	szPBP	S121	15	15	0	0.00	0.00
47367	szPBP	S121	15	15	0	0.00	0.00
35402	szPBP	S157	6	12	0	0.00	0.00
35402	PN/PIF	S145	3	70	12	80.00	14.63
47367	PN/PIF	S109	4	77	21	84.00	21.43
47319	PN/PIF	S122	3	34	11	78.57	24.44
47367	rmVTA	S145	103	6	3	2.83	33.33
35402	rmVTA	S169	139	7	9	6.08	56.25
47319	rmVTA	S145	94	16	3	3.09	15.79
35402	SuM	S169	143	2	8	5.30	80.00
47319	SuM	S145	178	3	3	1.66	50.00
47367	SuM	S145	178	1	7	3.78	87.50
35402	RLi	S145	17	3	5	22.73	62.50
47367	RLi	S109	26	10	14	35.00	58.33

Vglut2			absolute cell counts			% coexpressing	
animal	area	section	Tomato only	Vglut2 only	coexpressing	of tomato	of Vglut2
35402	IF	S132	12	13	10	45.45	43.48
35402	IF	S144	10	13	10	50.00	43.48
47367	IF	S104	12	18	12	50.00	40.00
47367	IF	S116	5	10	5	50.00	33.33
47319	IF	S112	5	12	6	54.55	33.33
47319	IF	S124	4	11	5	55.56	31.25
35402	PBP	S144	10	15	4	28.57	21.05
47367	PBP	S116	12	82	10	45.45	10.87
47369	PBP	S112	7	54	9	56.25	14.29
47369	szPBP	S124	1	7	8	88.89	53.33
47367	szPBP	S116	1	15	12	92.31	44.44
35402	szPBP	S156	1	11	14	93.33	56.00
35402	PN/PIF	S144	7	6	5	41.67	45.45
47367	PN/PIF	S116	19	14	12	38.71	46.15
47319	PN/PIF	S116	10	8	6	37.50	42.86
47367	rmVTA	S152	27	150	75	73.53	33.33
35402	rmVTA	S168	43	139	89	67.42	39.04
35402	SuM	S168	43	42	82	65.60	66.13
47367	SuM	S152	51	139	114	69.09	45.06
35402	RLi	S144	7	31	20	74.07	39.22
47367	RLi	S116	11	45	22	66.67	32.84

Viaat			absolute cell counts			% coexpressing	
animal	area	section	Tomato only	Viaat only	coexpressing	of tomato	of Viaat
35402	IF	S138	16	8	3	15.79	27.27
47367	IF	S101	18	12	5	21.74	29.41
47367	IF	S113	11	5	2	15.38	28.57
47319	IF	S113	11	9	4	26.67	30.77
35402	PBP	S138	11	111	5	31.25	4.31
47367	PBP	S101	26	103	8	23.53	7.21
47367	PBP	S113	16	110	3	15.79	2.65
47369	PBP	S101	8	NC	4	33.33	NC
47369	PBP	S113	14	87	8	36.36	8.42
47369	szPBP	S113	13	13	1	7.14	7.14
47367	szPBP	S113	9	12	1	10.00	7.69
47367	szPBP	S125	5	7	1	16.67	12.50
35402	PN/PIF	S138	21	12	4	16.00	25.00
47367	PN/PIF	S113	18	12	5	21.74	29.41
47367	rmVTA	S149	100	1	0	0.00	0.00
47319	rmVTA	S149	92	9	2	2.13	18.18
47319	SuM	S149	95	52	75	44.12	59.06
47367	SuM	S149	152	65	69	31.22	51.49
35402	RLi	S138	21	9	3	12.50	25.00
47367	RLi	S113	26	9	3	10.34	25.00
